# An Improved Free Energy Perturbation FEP+ Sampling Protocol for Flexible Ligand-Binding Domains

**DOI:** 10.1038/s41598-019-53133-1

**Published:** 2019-11-14

**Authors:** Filip Fratev, Suman Sirimulla

**Affiliations:** 1 Department of Pharmaceutical Sciences, School of Pharmacy, University of Texas at El Paso, El Paso, TX 79968 USA; 2 Micar21 Ltd., Persenk 34B, 1407 Sofia, Bulgaria

**Keywords:** Structure-based drug design, Computational chemistry

## Abstract

Recent improvements to the free energy perturbation (FEP) calculations, especially FEP+ , established their utility for pharmaceutical lead optimization. Herein, we propose a modified version of the FEP/REST (i.e., replica exchange with solute tempering) sampling protocol, based on detail studies on several targets by probing a large number of perturbations with different sampling schemes. Improved FEP+ binding affinity predictions for regular flexible-loop motions and considerable structural changes can be obtained by extending the prior to REST (pre-REST) sampling time from 0.24 *ns/λ* to 5 *ns/λ* and 2 × 10 *ns/λ*, respectively. With this new protocol, much more precise ∆∆*G* values of the individual perturbations, including the sign of the transformations and decreased error were obtained. We extended the REST simulations from 5 *ns* to 8 *ns* to achieve reasonable free energy convergence. Implementing REST to the entire ligand as opposed to solely the perturbed region, and also some important flexible protein residues (pREST region) in the ligand binding domain (LBD) has considerably improved the FEP+ results in most of the studied cases. Preliminary molecular dynamics (MD) runs were useful for establishing the correct binding mode of the compounds and thus precise alignment for FEP+ . Our improved protocol may further increase the FEP+ accuracy.

## Introduction

Accurate *in silico* predictions of ligand-protein binding affinities continues to be a primary objective of structure-based pharmaceutical design because of its putative value for drug discovery. Improvements to binding affinity and selectivity are critical to hit-to-lead optimization efforts. Free energy perturbation (FEP) calculations are attractive for predicting ligand-protein binding affinities via molecular simulations as well as for reducing the duration of the lead optimization phase of pharmaceutical development, which is as an individual stage the most expensive part of drug discovery^1,2^. Due to increased graphics processing unit (GPU) computational power the applications of FEP, especially FEP+ , has recently become very popular in both conventional lead and fragment optimization^3–7^. In general, FEP+ shows considerable correlations between calculated and experimental binding free energies, and average errors in the range of only 1 *kcal/mol*^3^. FEP calculations are based on molecular dynamics (MD) simulations and therefore explicitly consider both enthalpy and entropy effects of the conformational flexibility of the ligand, as well as desolvation effects within the ligand binding domain (LBD) of certain receptors. However, to date FEP has typically been helpful mainly when (1) high-quality X-ray data is available and (2) the target protein does not undergo significant conformational changes. Also, a lack of detail studies on determining an adequate sampling time is often one of the primary limitations of FEP calculations.

Further, FEP remains insufficient in several aspects and requires improvement. Detection of the most likely ligand binding mode, the presence of multiple stable binding conformations, insufficient equilibration, and determining an adequate sampling time (especially when significant protein side chain and backbone residue flexibility is possible) are the most critical aspects of FEP, and have been recently reviewed in detail^8^. Although these problems can be at least partially resolved by execution of reasonably long (≈100–300 *ns*) preliminary MD simulations, such an approach is often underutilized in applied simulations^8–10^. Many failures of FEP calculations are attributable to underestimating the importance of the preliminary MD studies^8^. Recently, we proposed a common computational workflow which can retrieve accurate information about the ligand binding modes and the starting pose for FEP+ simulations^11^. We recommend using this workflow before setting up and executing FEP+ calculations, especially for flexible protein structures. Moreover, knowledge about protein dynamics, such as identifying residues that are critical to ligand binding within the LBD, may further improve the FEP+ protocol; for example, by including these residues in the replica exchange solute tempering (REST) region (also known as a “hot region”, where more detailed sampling is made (protein REST (pREST region))^12,13^. Thus, the preliminary MDs can also provide a guide for developed pREST methodology^14^ which aims to resolve critical structural rearrangements. If multiple binding poses are known for a series of compounds, calculations can be set up to rigorously evaluate their contributions to the binding free energy^15^. Starting in the correct configuration is advantageous because free energy calculations are more sensitive to protein conformation relative to traditional structure-based drug design tools such as docking, where only binding site residues contribute significantly to the results^8^. It is also important to well-equilibrate the starting system of interest and provide the optimal starting point for free energy calculations^8,11,16^.

The sampling time of FEP calculations is another key issue^8^. In order to reduce the computational time and avoid unrealistic conformational states several FEP sampling protocols have been developed. Most of these protocols employed similar short-term sampling simulations schemes which in many cases were incapable to provide reasonable results^8,17^. However, to our best knowledge there are no detail studies on the necessary sampling time of FEP calculations^8^. In an attempt to improve the FEP+ results most studies focused only on exploring the REST procedure sampling times but not the pre-REST step. For instance, the effect of longer FEP/REST simulations was explored, showing average errors decreasing from 0.9 to 0.6 *kcal/mol* for a series of beta-secretase 1 (BACE1) systems when REST simulations times were increased from the default time of 5 *ns* to 20 *ns/λ*^18^. Furthermore, extending the REST simulations for a series of c-Jun N-terminal kinase 1 (JNK1) ligands from 5 *ns* to 10 *ns* per replica improved the average absolute energy difference from 0.7 to 0.4 *kcal/mol*^3^. However, increasing the REST time does not always guarantee accurate predictions^8,17^.

In this study, we performed detail studies on several targets to obtain and adjust the best possible sampling times for FEP+ calculations. Here, we propose a modified version of the FEP+ sampling protocol created by probing a large number of perturbations with different sampling schemes. Our sampling protocol can be divided into two sub-protocols which are useful in different cases: (1) the 5-*ns* pre-REST and 8-*ns* REST simulation protocol typically provides reasonable results when either an X-ray structure is available or there are no significant structural rearrangements, whereas (2) the 2 × 10-*ns* (two independent 10-*ns* runs) pre-REST sampling per lambda is more suitable for systems in which there are significant structural changes. The first sampling protocol only relaxes the system, enabling the ligands to adopt a reasonable equilibration and conformation, whereas the second one is structurally independent and can assist in describing the transition between some free energy minima, in terms of both ligand and protein conformations. We have developed our FEP+ modified protocol based on one ligand-protein system and then tested it on four different protein systems.

## Methods

Four test systems were examined in detail in our current study. Two of these sets (THR and TYK2) were also used by Schrödinger Inc. in their initial FEP+ validation and one (T4 lysozyme L99A) for pREST methodology. We used the same sets of ligands and compared the results to the work of Wang *et al*.^3^ (Supporting information Excel sheet) and Lim *et al*.^14^ (Supporting information Table 4 to 8). In addition, a set of AKT1 kinase inhibitors was also employed as a test system. Our improved sampling FEP+ protocol was initially developed based on a series of PPAR gamma partial agonists but only the results from the selected test systems are present in the main text of the paper. Additional details on the protocol development and motivation of the study, including PPARγ case, is provided in Supporting information (SI).

### Protein and ligands preparation

Protein structures were downloaded from the Protein Data Bank (pdb; (www.rcsb.org). For the PPAR*γ* study both the DA (pdb id 3U9Q) and Rosiglitazone (pdb id 1FM6) structures were used during the calculations e.g. for the initial development of our protocol. Docking calculations to place compounds into the PPAR*γ* ligand binding domain (LBD) were performed with Glide version 6.4 (Schrödinger 2017–3)^19^ with default parameters in a XP docking mode. Protein structure with pdb id 3QKK^20^ was employed for the AKT1 studies and the ligands were aligned into the compound represented in the X-ray structure. All other structures were the same as in refs^3,14^ in order a best possible comparison of the sampling protocols to be performed. Thus, for T4 lysozyme L99A, THR and TYK2 the structures with PDB ids 4W52 (the closed state conformation), 2ZFF and 4GIH were employed. We used also the same alignment as in the aftermentioned studies. Note that for THR and TYK2 we used the input files supplied by Wang *et al*.^3^. All missing residues were build up by the protein refinement tool included in Schrodinger 2017–3 package. All of them are in the loop parts and are far away from the studied binding pockets. They have negotiable contribution to the calculated binding energies.

The X-ray structure preparation for subsequent modelling was conducted with the Protein Preparation Wizard^21^. Missing atoms and H-bond network by assigning tautomer/ionization states, sampling water orientations and flipping Asn, Gln, and His residues in the plane of their pi-systems were optimized assuming a pH = 7.0. All resolved crystal water molecules were maintained. Ligand 3D-structures where sketched manually and transformed into low-energy 3D-structures using ligprep version 3.5.

### Selection of sampling times

Initially, we found the best sampling approach by execution of many combinations of sampling times using two types of MD derived PPAR gamma structures (averaged and from clustering). Thus, in the first case the structure will eventually need more sampling time, because it represents several energy minima combined, whereas in the second case it represents only the most populated state. To explore FEP+ sampling protocols which can provide optimal results, we performed a detail study focusing not only on REST but also the prior to REST (pre-REST) component of the FEP+ computational workflow. To obtain adequate sampling times for both the pre-REST and REST simulations and standardize our protocol, we probed a significant number of combinations of different per-lambda simulations. For the pre-REST simulations, instead of the default value of 0.24 *ns* per lambda (ns*/λ*), we used longer times and runs: 2, 5, 10, and 2 × 10 *ns/λ*. Relaxation times were maintained at default values. To our best knowledge, there are no detail studies on how pre-REST sampling simulations impact the final free energy predictions, convergence, and standard errors. We also varied the REST simulations between five windows (2, 5, 10, 20, and 50 *ns/λ*). Each of the pre-REST sampling times were combined with the remaining REST simulations and the results from all 20 combinations were analyzed. The convergence for each of these combinations was closely monitored. We paid special attention to ligand transformations that have a high (2–3 *kcal/mol*) free energy difference (∆∆*G*) and lower structural similarity. Data from such perturbations is limited and FEP+ has been primarily tested mainly on transformations with experimentally known ∆∆*G* values that are less than 1.5–2.0 *kcal/mol*. In addition to varying the sampling simulation times, we also explored the ligand portion that is suitable for inclusion in the “hot region” e.g. only the perturbation atoms or the whole structure. For some of our systems (e.g., T4 lysozyme and AKT1) how the inclusion of the protein residues impacts the FEP+ results was examined (pREST methodology).

### FEP+ calculation

All calculations have been conducted using the Schrödinger molecular modeling suite 2017–3^19^. Free energy perturbation calculations were carried out using the FEP+ methodology, which combines the accurate modern OPLS3 force field^22^, GPU-enabled high-speed molecular dynamics simulations with Desmond version 3.9, the REST algorithm for locally enhanced sampling^12^, a cycle-closure correction to incorporate redundant information into free energy estimates, and the FEP Mapper tool to automate setup and analysis of the calculations. Note that our version of the Lead Optimization Mapper (LOMAP) (Schrödinger 2017–3) linkage of ligand permutations typically differed from those previously obtained, and thus to have reliable comparisons we used the same perturbations in most of the cases. However, for some of the systems we also show the default outputs of LOMAP. The force field builder tool was used to test if accurate OPLS3 force field torsional parameters for all molecules were available. Missed parameters for some of the PPAR*γ*, Thrombin and TYK2 inhibitors were calculated by additional QM calculations and fitting using the ffbuilder module. We report theoretical error estimates based on standard deviation of repeat simulations, and compare with experiment via the correlation coefficient (*R*^2^) and the mean unsigned error (*MUE*). *MUE* s are also reported with 99% confidence intervals.

**Figure 1:**
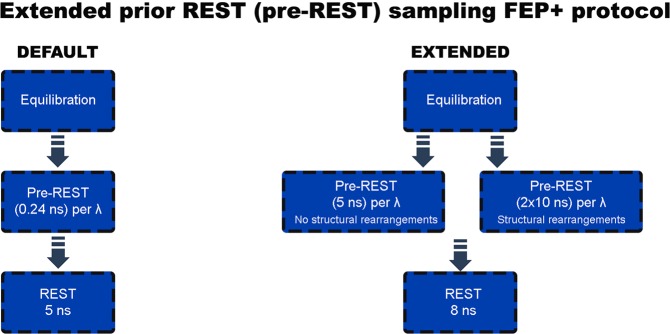
A flowchart which represents the steps of suggested new sampling protocol and comparison with the default one.

The FEP+ calculations based on the aftermentioned X-ray structures were conducted using developed new FEP+ sampling protocol but in case of PPAR*γ* and AKT1 we also employed the default protocol in order to compare the different workflows. In both cases the systems were solvated in an orthogonal box of SPC water molecules with buffer width (minimum distance between box edge and any solute atom) of 5Å for the complex and 10Å for the solvent simulations. For systems with net charge different than zero, counterions were included to neutralize the system with additional Na+ and Cl− ions added to achieve 0.15 M excess to mimic the solution conditions of the experimental assay. The full systems were relaxed and equilibrated using the default Desmond relaxation protocol, consisting of an energy-minimization with restraints on the solute, then 12 *ps* length simulations at 10 *K* using an NVT ensemble followed by an NPT ensemble. After that the restrained system was equilibrated at room temperature using the NPT ensemble. Finally, a 240 *ps* room temperature NPT ensemble simulation was conducted in a case of default FEP+ protocol. As we described in aforementioned details above for our sampling protocol, we used 5 *ns* and 2 × 10 *ns* pre-REST simulations. REST simulations were in the NPT ensemble and lasted 5 *ns* and 8 *ns*, for both the complex and the solvent systems, in the default protocol and our sampling scheme, respectively. For the transformations in solvent the default 5 *ns* REST simulation was employed but the pre-REST runs were with equally to the complex legs 5 or 2 × 10 *ns* - long simulation periods. Unless otherwise stated, the REST region only included heavy atoms of the ligand. A total of 12 *λ* windows were used for all FEP/REST calculations except in a case of some of the AKT1 ligands where the transformations included core-hoping FEP+ protocol, which consist of 16 *λ* windows. Replica exchanges between neighboring *λ* windows were attempted every 1.2 *ps*. For a more detailed description of the free energy calculation protocol employed, consult the Supporting Information of Wang *et al*.^3^. Figure 1 represent in schematic way our sampling protocol and a comparison with the default one. In some cases, as for example T4 lysozyme L99A and AKT1 systems, we included some of the protein residues to the “hot region” which we call here “pREST region” and is the same to those already defined in ref.^14^. For T4 lysozyme L99A these are Glu108, Val111 and Gly112 and for AKT1 were Gly159, Phe161 and Gly162. Note that these residues were included only in some of the simulations.

The convergence was closely monitored. No significant changes in the free energy were observed after 3 *ns* and 5 *ns* simulation time for templates derived from X-ray analyses and MD generated structures, respectively. More details and discussions are found in the FEP+ section of Results and Discussion section. All calculations were run on Nvidia Pascal architecture GPUs. An example of modified FEP+ input (*.msj) executable script is provided in (SI).

### Preliminary MD simulations

For some of the systems such as AKT1 and PPAR*γ* preliminary MD studies were performed. For these MD runs Amber 16 software^23^ were used. In Amber 14 and later versions, a new Monte Carlo (MC) barostat has been implemented in pmemd.cuda, which speeds up calculations up to 20% and is considered to be more rigorous. However, as we previously observed^11^ different ligands dynamics behavior was recorded between MC and Berendsen barostats^24^. We warned recently that the Berendsen barostat seems to be more suitable for ligand dynamics description^11^.

One possible explanation of this is that the pmemd.cuda does not compute the virial when using the MC Barostat. As a consequence of these observations, the Amber14SB FF^25^ was used along with Berendsen barostat in all the Amber 16 MD. simulations presented in the work. Complexes were immersed in a truncated octahedron box of TIP3P^26^ water molecules with a margin distance of 12.0 Å. Initially, the systems were energy minimized in two steps. First, only the water molecules and ions were minimized in 6000 steps while keeping the protein and ligand structures restricted by weak harmonic constrains of 2 *kcal/mol*^−1^ Å^−2^. Second, a 6000 steps minimization with the conjugate gradient method on the whole system was performed. Further, the simulated systems were gradually heated from 0 to 310 *K* for 50 *ps* (NVT ensemble) and equilibrated for 3 *ns* (NPT ensemble). The production runs were performed at 310 *K* in a NPT ensemble. Temperature regulation was done by using a Langevin thermostat with a collision frequency of 2 *ps*^−1^, and the pressure regulation via Berendsen barostats. The time step of the simulations was 2 *fs* with a nonbonded cutoff of 8Å using the SHAKE algorithm^27^ and the particle-mesh Ewald method^28^. For each of the studied ligand two independent 100 *ns* long simulations were executed. An exception was PPAR*γ*, in this system we executed 2 × 150 *ns* long MD runs.

## Results and Discussions

### T4 lysozyme (L99A): benzene derivatives

**Figure 2:**
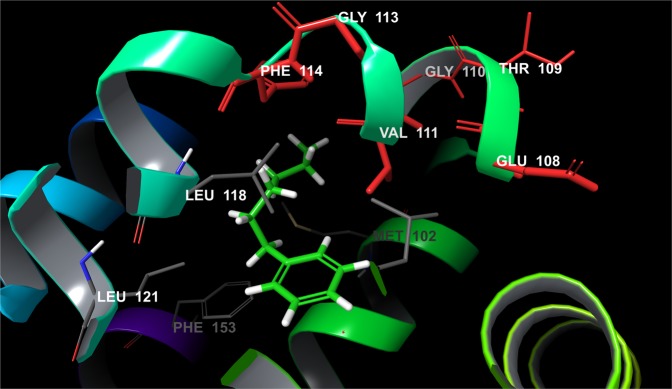
Structure of LBD of T4 lysozyme (PDB id 4W52) bond with Hexylbenzene. Residues from F-Helix (colored in red) are flexible and determine the LBD in an open, closed and intermediate state.

**Figure 3:**
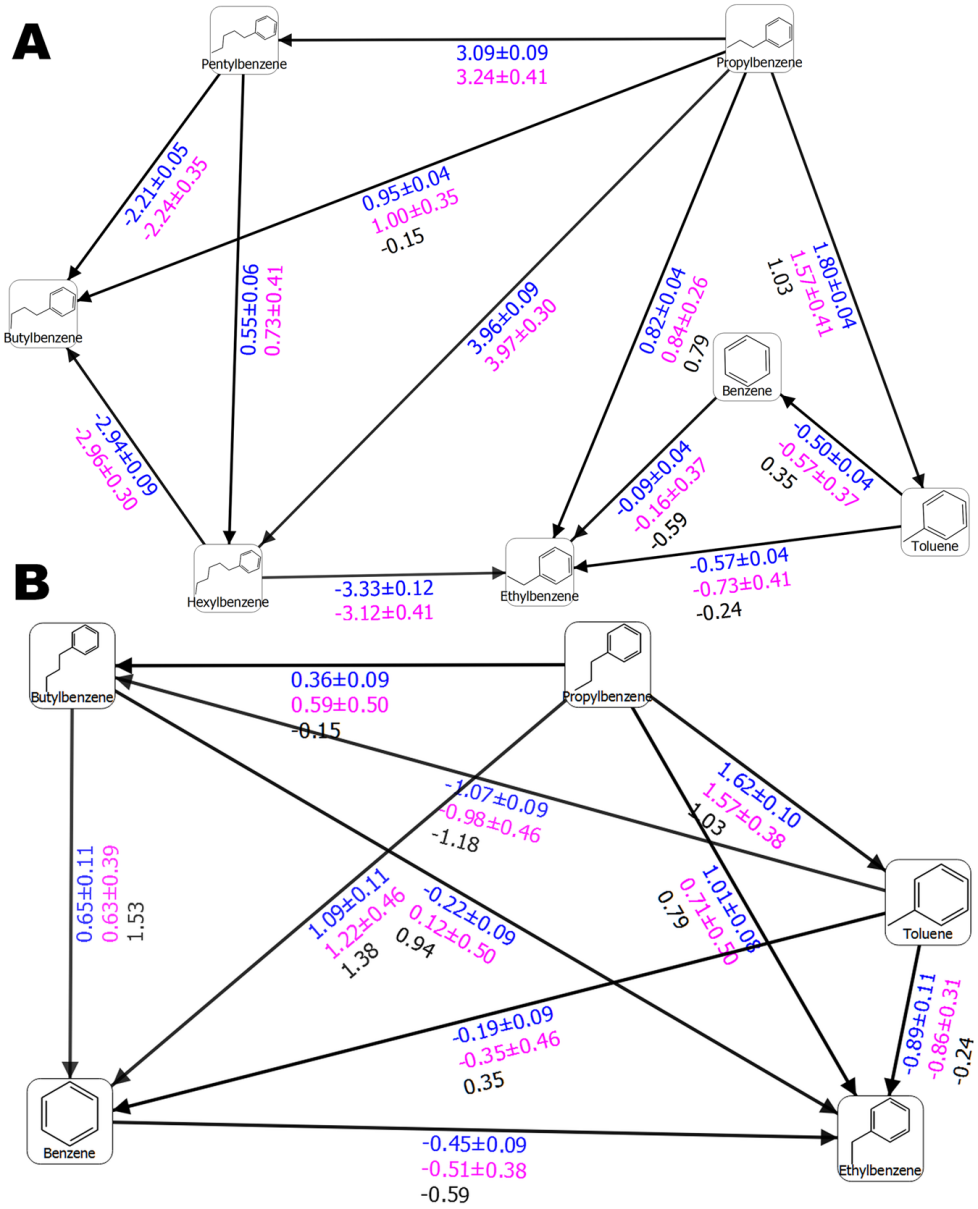
(**A**) Free energy output maps obtained via the 2 × 10 *ns* pre-REST FEP+ sampling protocol for all T4 lysozyme L99A test set of ligands and (**B**) for the compounds for which experimental ∆∆*G* values are available and the pREST protocol was also applied. Black, blue, and red numbers indicate the experimental (∆∆*G*_*exp*_), calculated Bennett (∆∆*G*_*pred*_), and cycle closure (∆∆*G*_*predc*_) free energies of binding, respectively.

An obvious example on which to test our sampling protocol for systems that undergo considerable structural rearrangements is T4 lysozyme L99A (Fig. 2), on which a detailed pertinent FEP+ study was recently reported^14^. Two different X-ray structures (in open and closed states) are available, yet conventional FEP+ simulations failed to reproduce the experimental data, due to inadequate sampling of both of these conformational states using a single structure. This was especially true for the transition between the so-called closed-open transformations. Our FEP+ calculations via the standard sampling protocol confirmed these results. To be structurally independent one sampling protocol must ensure the use of only one crystallographically resolved structure, because this is a limitation in many situations; e.g., only one Protein Data Bank (PDB) structure is deposited for the target system. We used as a reference the aforementioned study and accepted that the closed conformation is actually a deep energy minimum. Thus, we decided to use our 2 × 10 *ns* long pre-REST and 8-*ns*-long REST FEP+ sampling protocol along with closed conformation structure for all performed calculations. The perturbations were performed in a manner nearly identical to those in Lim *et al*.^14^ (Fig. 3A, Table S2A and Tables S4–6. Initially we focused on the primary issues detected in Lim *et al*.^14^.

Surprisingly, a ∆∆*G*_*pred*_ = 3.95 *kcal/mol* for the propylbenzene-to-hexylbenzene transformation was obtained, which was close to the previously calculated value of 3.38 *kcal/mol* via 55 *ns* FEP+/pREST approach. Considering that the default FEP+ and 5-*ns*-long pREST sampling protocols provided values of 5.85 and 4.93 *kcal/mol*, respectively^14^, we conclude that our sampling procedure well-predicted this transformation. Our simplified simulation protocol with 5-*ns* pre-REST and 8 *ns* REST sampling also provided a reasonable value (∆∆*G*_*pred*_ = 3.96 *kcal/mol*). A good result of ∆∆*G*_*pred*_ = 3.09 *kcal/mol* for propylbenzene to pentylbenzene perturbation was also obtained, whereas a value of ∆∆*G*_*pred*_ = 3.20 *kcal/mol* was reported in Lim *et al*.^14^. We also have some differences with the aforementioned paper. However, it is difficult to determine which results are more accurate without any available experimental data for these ligands. A careful examination of the MD trajectories showed that the longer pre-REST simulation time allows both the long side-chain ligands and the F-helix to adopt a reasonable conformation for the particular system. In an analogy to Lim *et al*.^14^, the F-helix conformational changes were described. This flexible portion sampled both the open and closed states to an almost equal extent depending on the ligand size (data not shown). The small-chain ligands were also well-described in most cases. This was especially true in examples for which the pREST procedure exhibited more difficulties. Examples include the transformation of propylbenzene to ethylbenzene (Fig. S2 and Tables S7–8 in Lim *et al*.^14^). Our protocol provided a value of ∆∆*G*_*pred*_ = 0.82 *kcal/mol*, which is nearly identical to the experimental value of 0.79 and an improvement compared to pREST simulations, ∆∆*G*_*pred*_ = 1.08 *kcal/mol*^14^. The prediction for propylbenzene-toluene perturbation (∆∆*G*_*pred*_ = 1.80, ∆∆*G*_*exp*_ = 1.03 *kcal/mol*) was also improved relative to those calculated by both the default FEP+ and FEP+/pREST sampling protocols: ∆∆*G*_*pred*_ = 2.21 and 1.91 *kcal/mol*, respectively. For toluene-ethylbenzene perturbation the pREST method provided a less-accurate value than standard FEP+ (−0.59 vs. −0.84 *kcal/mol*, ∆∆*G*_*exp*_ = −0.24), whereas our approach provided a ∆∆*G* value of −0.57 *kcal/mol*. For most of the transformations 5 *ns*-long REST simulation time was satisfactory in order to get a good convergence with some exceptions, such as the case of propylbenzene → benzene, where it is clear that at least 8 *ns* REST runs are necessary although the improvement is only 0.1 *kcal/mol* (Figs S3 and S4). Standard errors seem to affect our protocol to a lesser extent than other protocols and are more pronounced in pREST, probably due to the inclusion of protein residue atoms in the hot region.

The regular deviations in a frame of our sampling protocol were between 0.04 and 0.06 *kcal/mol*, whereas those produced by 5-*ns* pREST sampling were in the range of 0.08 to 0.16 *kcal/mol*, respectively. In summary, the standard cycle closure errors between our 2 × 10-*ns* pre-REST and previously used 55-*ns*-long pREST samplings were comparable.

However, in the case of butylbenzene we obtained a poor sampling of the intermediate F-helix state and therefore poor free energy predictions. Nevertheless, our *RMSE* value of 0.69 *kcal/mol* is still reasonable, and decreased to less than 0.5 *kcal/mol* after removing the butylbenzene outlier. This *RMSE* is between those obtained by Lim *et al*.^14^ via the standard FEP+ sampling protocol (1.0 *kcal/mol*) and the 55-*ns*-long pREST protocol (0.54 *kcal/mol*); i.e., still provides quite reasonable free energy predictions. Transformations generated by the default FEP+ Lead Optimization Mapper (LOMAP) tool provided fewer connections and there was only one leg present with butylbenzene. Consequently, the *RMSE* error via our sampling protocol decreased to 0.61 *kcal/mol*.

An explanation of the butylbenzene outlier is that the longer pre-REST sampling protocol can resolve structural rearrangements that are pertinent to significant conformational changes. The 2 × 10-*ns* pre-REST step assures an adequate simulation time for the transformation from one major state to another introduced by the ligand; i.e., an adaption of the system. For T4 lysozyme this is the closed to open F-helix conformations but intermediate state sampling, especially those for the ligand, is more pertinent to the REST simulations because of the increased ligand sampling via the replica exchange molecular dynamics (REMD) approach in the hot region. In all of the 2 × 10-*ns* pre-REST simulations, butylbenzene transforms the F-helix to its open state and we recorded an RMSD of nearly 3.0 Å for the compound, whereas T4 lysozyme bound to a short-chain ligand featured an RMSD of about only 1.0 Å (Fig. S5). This is further evident from the calculated ∆∆*G* value for the butylbenzene-to-benzene perturbation of 0.26 *kcal/mol*; similar to what was obtained via the standard FEP+ protocol for the open conformation, ∆∆*G* = 0.59 *kcal/mol*, but not the closed conformation, ∆∆*G* = −0.58 *kcal/mol* (Tables S7–8 in Lim *et al*.^14^). Thus, in contrast with the standard FEP+ sampling protocol, the poorly predicted values for this ligand arise not because the protein is in a closed conformation but due to a permanent transition to an open conformation, suggesting less sampling for the intermediate conformation. An another reason for butylbenzene’s suboptimal free energy predictions is that this compound has a unique conformation; the dihedral angle between the benzene ring and ethyl chain differs from those of analogous molecules. It can adopt values of either approximately 90° or nearly 150°. This conformation is due to the unique interactions with Val111 and improved sampling of these residues is necessary. Thus, to overcome the problem with butylbenzene we also included into the hot region the three amino acid residues from the F-helix (Glu108, Val111, and Gly113) in an identical manner to Lim *et al*.^14^ i.e., we mixed our sampling protocol with that of pREST. As a result the calculated ∆∆*G* values were equal to those using only the pREST method executed for 55 ns; Fig. 3B, Table 2B and Table S8 in Lim *et al*.^14^. The final results for all of the ligands with known experimental free energies were *RMSE* = 0.57, *MUE* = 0.48, and *R*^2^ = 0.64. Hence, including the pREST procedure enabled us to further improve our FEP+ calculations. This is a clear indication that our protocol can be used for sub-structural (helix/loop) reorganizations, independent of the starting structure, which was the closed L99A conformation in our case.

In summary, our enhanced sampling protocol significantly improves the results for T4 lysozyme compared to the default FEP+ sampling method (Table 1). The correlation coefficient for the relationship between ∆∆*G*_*pred*_ and ∆∆*G*_*exp*_ increased from *R*^2^ = 0.10 to *R*^2^ = 0.64 and the RMSE decreased from 1.04 to 0.57 for the default and our FEP+ sampling protocol, respectively. An obvious advantage is the reduced time necessary to perform the pREST FEP+ calculations. Based on the aforementioned results it is clear that researchers can obtain similar results for time periods that are 2 times shorter: 55 *ns* required for pREST and 22-28 *ns* required for our sampling protocol. For T4 lysozyme, using 4 × GTX 1080Ti GPUs, one perturbation necessitated approximately 7.5 h. It should be also noted that the above mentioned results in Lim *et al*.^14^ have been obtained by only averaging the last (44–55 *ns*) duration of the simulation, which also requires a more in-depth study of the system; this is unnecessary in our protocol.

**Table 1:** Direct comparison between the results obtained by the default FEP+ and our sampling protocols for each studied system.

	Default FEP+			Improved FEP+			
	RMSE	MUE	R^2^	RMSE	MUE	R^2^	n^**^
T4 lysozyme	1.04	0.88	0.10	0.57	0.49	0.64	10
AKT1	1.43	1.26	0.65	0.78	0.64	0.70	16
THR^*^	0.93	0.76	0.50	0.78	0.65	0.87	16
TYK2	0.93	0.75	0.79	0.58	0.41	0.86	24
PPARγ	3.33	2.73	0.82	1.86	1.51	0.97	9

^*^For T4, THR, TYK2 systems the data for the default protocol is from ref.^3^^,^^14^. ^**^n is number of perturbations.

### Series of AKT1 inhibitors

**Figure 4:**
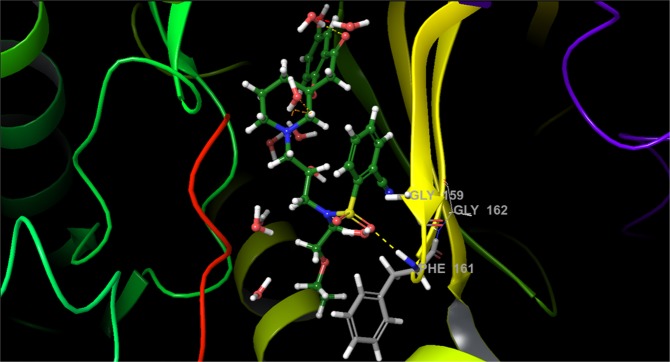
Structure of LBD of AKT1 (PDB id 3QKK) bond with ligand 18. The substrate peptide and F-loop are colored in red and yellow, respectively. The H-bonds are shown in yellow dot lines.

Further, we employed our FEP sampling protocol and compared the results to the default FEP+ sampling scheme on a series of known AKT1 inhibitors^20^ (Table 1). The AKT1 kinase exhibits both F-loop flexibility and differences in the ligand binding modes. The F-loop conformational diversity is quite visible in the resolved X-ray structures and it adopts a ligand-dependent conformation (Fig. 4). For a selected series of eight compounds, three compounds have been determined by X-ray crystallography when bound to the protein and exhibit different conformations, which clearly demonstrates that the ligand orientation in the binding cavity modulates the conformation of the ligand-loop system. In addition to the F-loop flexibility the LBD is exposed to solvent, populated with structurally important water molecules, and a natural peptide is also present which may also challenge FEP+ calculations. Moreover, as in the case of the PPAR*γ* receptor (see Supporting information), we also included permutations with low structural similarity in the range 0.2–0.4, which have been not broadly studied to date. Thus, we selected AKT1 for a detailed case study of our improved FEP+ sampling protocol.

Initially, we selected five compounds similar to ligand 21, which has been X-ray resolved^20^. We chose these ligands due their presumed identical binding modes. The default FEP+ sampling protocol gave RMSD = 1.23, *MUE* = 1.10 *kcal/mol*, and *R*^2^ = 0.05. Inclusion of residues Gly159, Phe161, and Gly162 (on top of the F-loop, and important to ligand-protein interactions) in the “hot region,” in accordance with the pREST protocol^14^, worsened the results to RMSE = 1.34, *MUE* = 1.13 *kcal/mol*, and *R*^2^ = 0.14 (Figs S6 and S7). Instead, our 5-*ns* pre-REST and 8-*ns* REST sampling protocol improved these numbers (*RMSE* = 0.84 and *MUE* = 0.74 *kcal/mol*). The primary improvement is due to the predicted positive value obtained for the transformation of ligand 19 to ligand 21, the sign of which becomes positive, in an agreement with experimental data (Fig. S8).

Neither protocol described the 18–15 perturbation to our satisfaction. Our extended sampling protocol with 2 × 10-*ns* pre-REST and 8-*ns* REST sampling provided the best results for the 18–15 ligand transformation. Although as per this simulation the Bennett error for the 18 to 15 perturbation was less than 1 *kcal/mol* it was still not well-described because the sign of ∆∆*G*_*pred*_ (+0.11 *kcal/mol*) differed from those of ∆∆*G*_*exp*_ (−0.84 *kcal/mol*). Moreover, the increased sampling times for each lambda also led to significantly more computational time. For the 2 × 10- *ns* and simplified 5-*ns* pre-REST sampling protocols the required computational times for each of these six perturbations, executed on a 4 × GPU GTX 1080Ti workstation, were 9 h and 2 h, respectively.

To understand the problems caused by ligand 18 and to verify whether MD-retrieved AKT1 structures can be used during FEP+ calculations, we ran a preliminary 100-*ns*-long conventional MD simulation of the 19-AKT1 complex via AMBER 14SBFF in the Amber 16 package. This ligand was chosen because it has the larger group on second position in the ring (CF_3_), which we believed can have the greatest impact on the ligand-loop flexibility. We assumed that a refined ligand binding mode would provide an optimal starting point for the FEP+ alignment and calculations because the substitutions at the second position in the ring, close to the F-loop, were common to the entire ligand series. Our averaged MD structure was nearly identical to the X-ray structure; the only considerable difference was in the ligand conformation, particularly the rotation of the phenyl ring. Thus, although ligand 21 (which has two methyl groups) is stabilized within the F-loop (as has been seen in the X-ray structure), such stabilization is not possible for the other substituted compounds as they exhibit slightly different binding modes.

**Figure 5:**
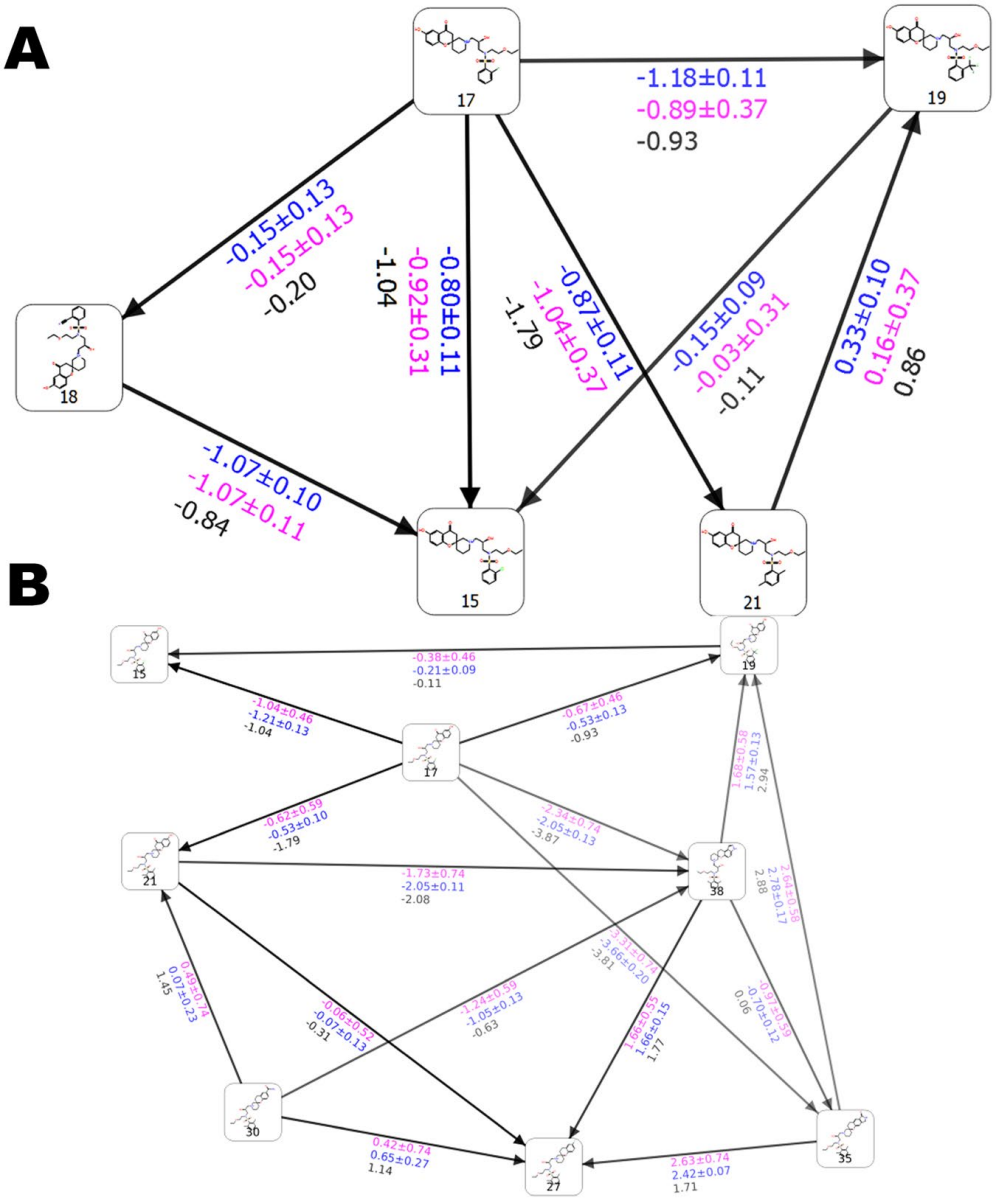
Free energy output maps obtained via the 5 *ns* pre-REST FEP+ sampling protocol for (**A**) selected small and (**B**) full test set of AKT1 ligands. Black, blue, and red numbers indicate the experimental ∆∆*G*_*exp*_, calculated Bennett (∆∆*G*_*pred*_), and cycle closure (∆∆*G*_*predc*_) free energies of binding, respectively.

To verify our hypothesis that MD-based refinement might be useful, we ran a new FEP+ simulation using the MD-derived 19-AKT1 complex and our extended 5 *ns* long pre-REST FEP+ sampling protocol. We greatly improved the ligand 19 transformations and overall results: *RMSE* = 0.46, *MUE* = 0.34, and *R*^2^ = 0.84 (Fig. S9), thus demonstrating that the modern force fields can produce high-quality structures which are suitable for FEP calculations, and pre-FEP MD preliminary studies should not be avoided as has been demonstrated in our recent work^11^. However, the perturbations of ligand 18 were still unimproved. Thus, we looked more deeply into this ligand and ran an additional 100-*ns*-long simulation of the 18-AKT1 complex, in the same manner as aforementioned for compound 19. Based on this MD run we revealed that the phenyl ring was flipped and the CN group was unable to adopt a conformation in an orientation forward into the F-loop; i.e., was rotated approximately 180 degree in the opposite direction. Hence, the correct position of this substituent during the initial FEP alignment was incorrect and the REST simulation, as in other cases^14,17^, was unable to identify this structural rotation. Thus, we aligned this substituent on position five in the ring, instead of position two. Thereupon, the FEP+ results were dramatically improved and the 18–15 perturbation was adequately described: ∆∆*G*_*pred*_ and ∆∆*G*_*exp*_ = −1.07+/−0.1 and −0.84 *kcal/mol*, whereas the *RMSE*, *MUE*, and *R*^2^ were 0.40, 0.28 *kcal/mol*, and 0.85, respectively (Fig. 5(A)). This lends additional support to our recent results^11^ that the MD preliminary studies are greatly beneficial for recovering the ligand binding modes and should be used for the FEP ligand alignment and calculations. Moreover, based on these results it is evident that the FEP+ extended pre-REST sampling as implemented in our protocol was insufficient to effectively sample these conformational states either due to the short simulation time or issues with the OPLS3 FF applied on proteins.

In summary, our results show that extended sampling significantly improved the FEP+ calculations and the correct binding mode recovery via preliminary MD simulations is critical to the FEP results. Using an MD-derived structure of AKT1 improved the FEP calculations. The 5-*ns* pre-REST and 8-*ns* REST simulations were sufficient to ensure good sampling and recovered the likely binding modes for most of the studied ligands, except in cases of ligand 18 where more-intensive (pre-FEP) MD sampling was required. Thus, we next concentrated on testing our 5-*ns*-long pre-REST protocol assuming that it will be able to handle the deviations in binding modes and structural changes in the protein starting from its X-ray structure. We removed ligand 18 but included three new compounds. Fifteen perturbations were included and compared to the default FEP+ sampling protocol. The crystal structures of 21, 27, and 38 showed slightly different binding modes yet increased activity due to structural modifications (∆∆*G*_*exp*_ = 2.94 *kcal/mol* for the 38 to 19 transformation), which also corresponded to decreasing ligand similarity.

Our FEP+ sampling protocol was well-compatible with these perturbations and predicted binding affinities were in a good agreement with experimental data: *RMSE* = 0.78, *MUE* = 0.64, and *R*^2^ = 0.70. The default FEP+ protocol did not perform as well (*RMSE* = 1.43, *MUE* = 1.26, and *R*^2^ = 0.65) and predicted with significant inaccuracy the 19–35, 35–27 (approximately 3 *kcal/mol* deviation), and 38–27 transformations, whereas calculated errors of ∆∆*G*_*pred*_ using our extended sampling scheme were quite low (Figs 5B and S10, Tables S3A and S3B). We observed an improvement for all of the calculated perturbations, which were most notable in transformations with large changes in binding activities. The sign of the activity change was correctly predicted in all of the transformations. Both the standard errors of the Bennett calculations and convergence were similar for standard and extended FEP+ (data not shown). This indicates that managing the binding conformations during the 5-*ns* pre-REST step of our protocol is the primary reason for the improvement in the FEP+ results. The extended simulation time well-sampled the structural changes in both the ligand and F-loop conformations.

A primary result of our sampling protocol for these AKT1 inhibitors was the correct predictions for compounds of low similarity and those featuring substitutions in three different structural positions. This gives us confidence in possibly expanding the application of our FEP+ extended sampling protocol to a series of more-diverse ligands.

### Thrombin inhibitors

**Figure 6:**
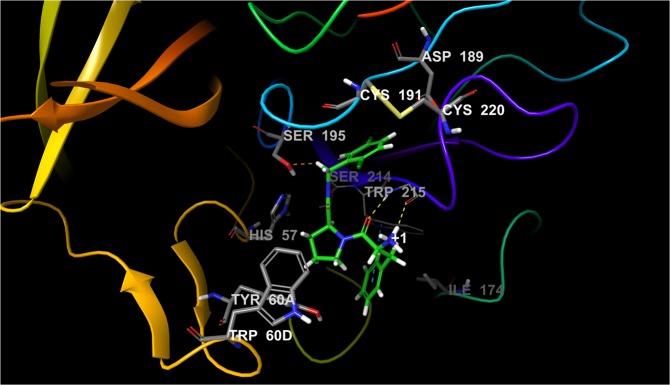
Structure of LBD of THR (PDB id 2ZFF) bond with ligand 1a. Ligand is rendered as green stick model and protein rendered as cartoon model. The H-bonds are shown in yellow dot lines.

Finally, we tested our protocol on some systems that are more-rigid and well-studied via FEP+ such as thrombin (THR) and tyrosine kinase 2 (TYK2)^3^. We chose the THR (Fig. 6) because it showed the lowest *RMSE* from all of the studied systems in Wang *et al*.^3^ and we were intrigued as to whether the *RMSE* could be further improved. The sampling protocol with 5-*ns* pre-REST and 8-*ns* REST was employed, which in accordance with AKT1 results seems to be compatible with both crystal structures and moderate structural changes. For both the THR and TYK2 ligand sets we used the automated FEP map builder LOMAP to set up the compound transformations, but only 38% of the perturbations for THR were identical to those in Wang *et al*.^3^. Thus, to compare our results to the default protocol we also included the same ligands and perturbations as in Wang *et al*.^3^.

Initially, we executed a small set of perturbations that have been not previously examined and for which higher deviations in the calculated ∆∆*G* values may be expected due to the large difference in the experimental free energies. After execution of the standard FEP+ protocol errors of more than 2 and 1.4 *kcal/mol* were obtained for the 6a-1a and 1a-1c transformations, respectively. We calculated *RMSE* = 1.54 and *MUE* = 1.44 *kcal/mol*. Our 5 *ns* pre-REST FEP+ sampling protocol improved the ∆∆*G* error of 6a to 1c transformation from 0.8 to 0.3 *kcal/mol*, and decreased the *RMSE* and *MUE* to 1.22 and 1.11 *kcal/mol*, respectively (see Figs S10 and S11). This data demonstrates that the difference in the results is not due to the force field: OPLS3 in our study and OPLS2.1 in Wang *et al*.^3^. The simulation time for one perturbation increased from 90 min to 200 min (5 *ns* pre-REST vs default FEP+) in our 8xGTX 1080Ti cluster, but for the modern generation of GPUs such as Pascal and Volta this is an acceptable range.

**Figure 7:**
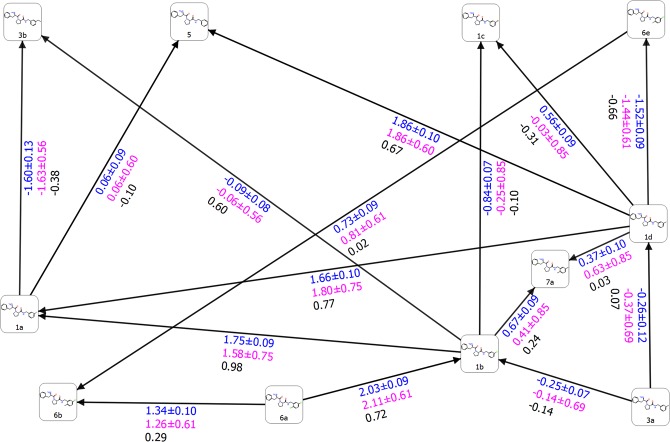
Free energy output map obtained via the 5 *ns* pre-REST FEP+ sampling protocol for the selected test set of THR ligands. Black, blue, and red numbers indicate the experimental (∆∆*G*_*exp*_), calculated Bennett (∆∆*G*_*pred*_), and cycle closure (∆∆*G*_*predc*_) free energies of binding, respectively.

Further, we executed the same perturbation as in Wang *et al*.^3^ and performed a direct comparison between the results obtained by the default and improved FEP+ sampling protocols (Fig. 7, Table S4 and SI Excel sheet in Wang *et al*.^3^). The *RMSE* and *MUE* values were improved: 0.93 to 0.78 and 0.76 to 0.65, respectively^3^. The *R*^2^ value greatly increased, from 0.5 to 0.87 (Table 1), proving improved predictive power of our sampling protocol. This was also made clear by a closely evaluating individual perturbations. The error for the 1d → 6e transformation decreased from 1.71 to only 0.78 *kcal/mol*. The same was obtained for the largest error calculated in Wang *et al*.^3^ :1.95 to 1.19 *kcal/mol*. We also achieved good convergence for the simulations (Fig. S12 and Table S4) and the cycle closure errors were less than 0.8 *kcal/mol*, which is an improvement of 0.4 *kcal/mol* compared to those obtained via default FEP+ calculations^3^. The ∆∆*G*_*pred*_ errors were less than 1.3 *kcal/mol* in all of these 16 executed perturbations.

For the entire THR set of ligands Wang *et al*.^3^ studied only the transformations with up to 0.8 *kcal/mol* difference in ∆∆*G*_*exp*_. In this case one can expect to also have small calculated ∆∆*G*_*pred*_ values via the standard protocol where researchers use only the unattached X-ray structure. However, although the final *RMSE* could be small the sign of the ligand transformations is also very important during the lead optimization process, especially for a limited set of ligands, and therefore is significant for the quality of FEP calculations. A correct prediction of whether one ligand will be more or less active is, or at least should be, a fundamental aim of FEP. For instance, for the 1b-7a perturbation (∆∆*G*_*exp*_ = 0.24) both our and standard FEP+ sampling protocols gave an error of approximately 0.45 *kcal/mol*, yet our protocol calculated a ∆∆*G* value of +0.67 *kcal/mol*, whereas the standard protocol provided ∆∆*G* = −0.23 *kcal/mol* [SI Excel sheet in Wang *et al*.^3^]. This difference (as in the most of the remaining cases) was not due to extending the REST simulation time to 8 ns, which is clear from the convergence graph (Fig. S13), but is due to the 5-*ns* pre-REST simulations. Another example is the 1a–3b perturbation for which the standard and our workflows calculated ∆∆*G*_*pred*_ values of +0.69 and −1.60, respectively, whereas the experimental ∆∆*G* was −0.38 *kcal/mol*. In summary, using standard FEP+ the sign was correctly predicted for only 50% of the perturbations versus 75% via our sampling protocol.

FEP+ predictions based on the default LOMAP perturbation map and our sampling protocol was also satisfactory. In this case we obtained transformations with higher experimentally observed free energies, yet we achieved similar results (*RMSE* = 0.74, *MUE* = 0.64, and *R*^2^ = 0.74; Fig. S14). The combined results from all of the calculations in one map are also shown (Fig. S15).

### TYK2 inhibitors

**Figure 8:**
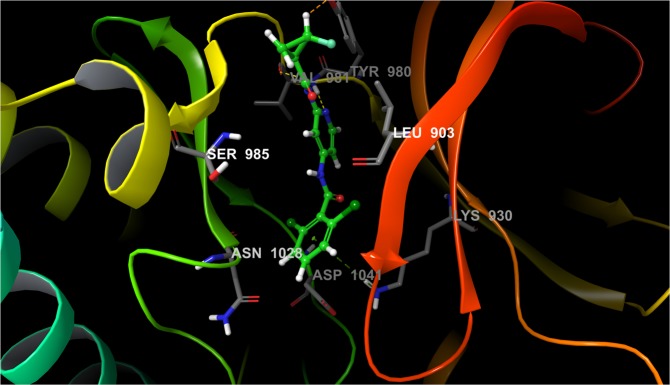
Structure of LBD of TYK2 (PDB id: 4GIH) bond with ligand ejm_46. The H-bonds are shown in yellow dot lines.

**Figure 9:**
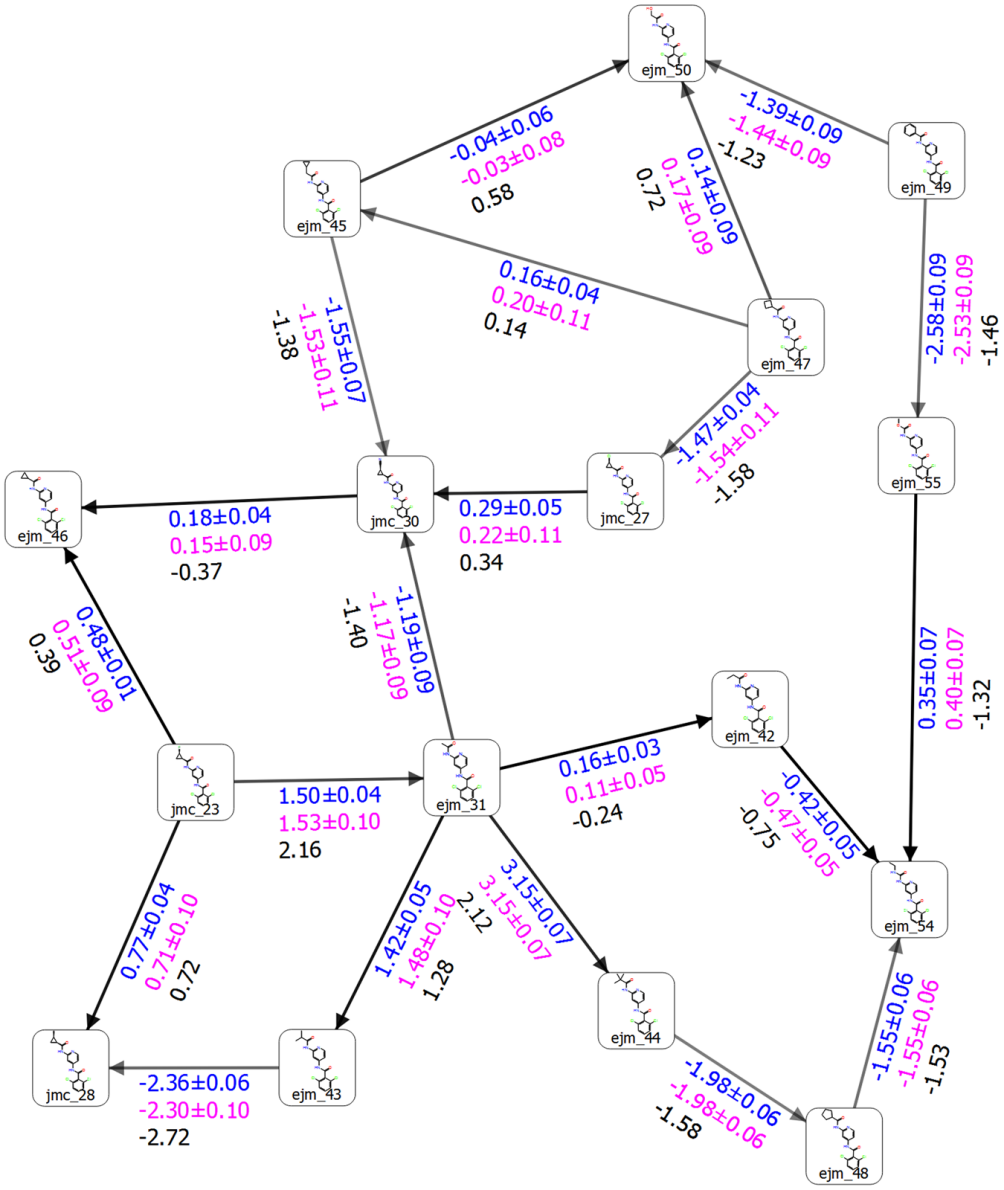
Free energy output map obtained via the 5 *ns* pre-REST FEP+ sampling protocol for the selected test set of TYK2 ligands. Black, blue, and red numbers indicate the experimental (∆∆*G*_*exp*_), calculated Bennett (∆∆*G*_*pred*_), and cycle closure (∆∆*G*_*predc*_) free energies of binding, respectively.

Finally, we also tested our new sampling protocol on TYK2 kinase. For this test set of inhibitors we again included the same ligands as in Wang *et al*.^3^ to compare the sampling FEP+ protocols (Figs 8 and 9). However, only the default LOMAP generated perturbations, most of which were same as in Wang *et al*.^3^, was used for the calculations. In this manner the number of transformations was reduced from 24 to 21, compared to those in Wang *et al*.^3^, which considering the THR results should not greatly affect the comparison to the regular FEP+ sampling method. Moreover, it is important to have more data from the default FEP map generation scheme, which is a common choice for typical users. Our 5-*ns* pre-REST and 8-*ns* REST sampling approach yielded quite low standard errors of the ∆∆*G* predictions, which were commonly less than 0.06 *kcal/mol* and 0.1 *kcal/mol* for Bennett and cycle closure results, respectively. This is approximately 4 × less than the regular FEP+ sampling protocol; Table S5 and SI Excel sheet in Wang *et al*.^3^ We found a significant improvement in the *RMSE* and *MUE* values, which decreased from 0.93 to 0.58 and 0.75 to 0.41 using the default FEP+ and our sampling protocols, respectively (Table 1). The correlation coefficient for the relationship between ∆∆*G*_*pred*_ and ∆∆*G*_*exp*_ increased from *R*^2^ = 0.79 to *R*^2^ = 0.86, demonstrating that in this system the prediction capability was also higher (Fig. S16). An improvement of the individual transformations presented in this study and those published in Wang *et al*.^3^ was also observed: approximately 0.3 *kcal/mol* for each perturbation.

In summary, we correctly predicted the sign of 81% of the transformations, comparable to standard FEP+ (79%). The lower standard errors are probably due to the effective pre-equilibration of each lambda before the more-extensive sampling of the REST procedure. We identified ligand ejm_55 as an outlier via both standard and enhanced FEP+ sampling protocols. The higher RMS error in standard FEP+ is in part due to the additional transformations of the ejm_55 ligand included in Wang *et al*.^3^ (presumably due to the efforts of Wang *et al*. to eliminate this outlier).

The most likely reason for the errors in the predicted free binding energy of ejm_55 is the different possible binding modes of this ligand, as in the case of the AKT1 test set ligands. We suspect that we can solve this issue via preliminary 100 to 200-*ns* MD simulations.

## Conclusions

We executed FEP+ calculations using many perturbations between a series of test ligands in several systems to determine optimal sampling times for the pre-REST and REST components of FEP+ simulations. Based on the obtained results it can be concluded that for more significant protein structural changes the longer sampling time is indeed a better option and our results showed that the optimal time is 2 × 10 ns pre-REST and 8 ns REST runs. It was meticulously chosen based on the combination of many sampling schemes. For the system with small structural deviations, introduced by the ligands, we found out that a significant improvement can be achieved only by 5 ns pre-REST sampling time. Thus, in the case of T4 Lysozyme, where some significant structural changes were introduced by the ligand, we applied our 2 × 10 *ns* pre-REST sampling protocol and for the rest of the systems the 5 ns one. The convergence was similar in both of the cases, but not the FEP+ accuracy. Based on this fact one can conclude that the convergence is not the key factor in our calculations instead it is the initial stage of the system, i.e. those before the REST procedure.

During developing our sampling protocol, we also performed directed comparison between our proposed and default sampling protocol with the same force field (OPLS3) for three of five systems. This also include one of the systems from the study of Wang *et al*.^3^. Thus, observed improvement is mainly due to the adjusting of the sampling times. We emphasize that not all of the extended sampling combinations provided improved results and in some cases they were much worse. For instance the 2-*ns* pre-REST was insufficient for AKT1, PPAR*γ*, and T4 lysozyme, and also for MD-derived averaged structures. We observed the same for the REST simulation time. We showed here that the starting system need not be the X-ray conformation. However, the pre-REST simulations must be extended to 5 *ns* rather than the default FEP+ sampling.

One of the most significant advantage of our protocol is that it provides FEP+ calculations in a structure-independent manner, similarly to the pREST approach^14^ but for a much reduced simulation time. Our sampling workflow can be used either for significant structural rearrangement or for conventional systems. It can be also applied to situations involving multiple ligand binding modes, and there are no restrictions on applying our approach in a combination with recently developed pREST methodology^14^. However, selection of the pREST region is not always obvious and does not necessary improve the results. Our data showed controversial results. Whereas for T4 lysozyme we obtained some improvement of the calculated free energies for the AKT1 system the results were much worse. Thus, the hot region is not a key part of our protocol. Instead, as for example for the T4 lysozyme we showed that the increased sampling of the prior REST FEP+ calculations (pre-REST) leads to unnecessary of the inclusion of protein residues in the hot region (pREST approach) and/or the time needed for the calculations employing our protocol is about 2 times less than the pREST method. Notably, according to the presented results we cannot see any significant difference in both the default and our FEP+ sampling protocols in the description of the Van der Waals (mostly presented in T4 lysozyme) and H-bond (AKT1 and THR systems) interactions.

Our proposed sampling protocol improves FEP+ in all cases, by values in a range of 0.3–0.8 *kcal/mol*, often by more than 1 *kcal/mol*. Significant improvements, including predicting the correct sign of the transformations, for more-complicated perturbations featuring a large difference in free energies was observed. This modified FEP+ protocol can be successfully applied to MD-derived structures (very important to homology modeling improvement and other applications) and provides improved results in all of the scenarios. We recommend this approach and anticipate that it will be widely adopted within the computational chemistry community.

## Supplementary information

Supporting Information

Dataset 1

Dataset 2
