# Supplementary material for: An Improved Free Energy Perturbation FEP+ Sampling Protocol for Flexible Ligand-Binding Domains
Source: Sci Rep. 2019 Nov 14;9:16829. doi: 10.1038/s41598-019-53133-1 (PMC6856381; doi:10.1038/s41598-019-53133-1)
Supplement: Supplementary file 1 — Supporting Information [file 41598_2019_53133_MOESM1_ESM.pdf]

Supporting Information

An Improved Free Energy Perturbation FEP+ Sampling Protocol for Flexible Ligand-Binding Domains

Filip Fratev[1, 2] and Suman Sirimulla[1]

[2]Department of Pharmaceutical Sciences, School of Pharmacy; University of Texas at El Paso, USA
[2]Micar21 Ltd., Persenk 34B, 1407, Sofia, Bulgaria

October 13, 2019

Contents

1 Motivation and Protocol Development **2**

2 Tables **3**

3 Employed scripts **4**

4 Figures **6**

References **23**

1 Motivation and Protocol Development

An initial motivation for this study came from our recent work in which we faced significant problems achieving accurate FEP+ results using MD-derived structures. We thus decided to find the best FEP+ sampling protocol that can provide us reasonable free energy predictions [1]. This protocol was developed based on a series of perfluoroalkyl acid (PFAA) ligands that bind to the PPARγ receptor, and in particular used the Perfluoroundecanoic acid (PFuDA)–PPARγ MD-derived structures. Initially, we used an averaged PFuDA–PPAR MD structure obtained by 2×150 ns MD simulation runs in Amber 16 software (see Methods) as a baseline for our FEP+ calculations using six ligands and nine perturbations. Using this MD-derived structure and the default FEP+ protocol provided exceptionally poor results even though the structure reasonably resembled the Decanoic acid (DA)–PPARγ resolved X-ray complex. We obtained an $RMSE$ of more than 3 $kcal/mol$, errors of 8 $kcal/mol$, and almost all of the perturbations showed a deviance of approximately 2 $kcal/mol$ compared to experimental data (see Figure S8 in ref [1]).

To find the optimal protocol for our set of ligands for PPAR we ran 2, 5, 10, and 2×10 ns/λ pre-REST simulations, in the context of FEP+, instead of the conventional 0.24 ns/λ. We carefully monitored the energy convergence and used 2, 5, 10, 20, and 50 ns/λ long REST simulations during our tests. System convergence in a solute was also monitored. We paid special attention to transformations that had a high (2–3 $kcal/mol$) free energy difference ($\Delta\Delta G$) and lower structural similarity. The results from standard FEP+ were used for reference. Execution of these 20 combinations, which were 180 perturbations in total, lead us to conclude that for an averaged MD structure a longer pre-REST simulation time of 2× 10 ns is optimal for obtaining reasonable results ($RMSE = 1.86$, $MUE = 1.51$, $R^2 = 0.97$). All perturbations featured an improvement via our 2×10 ns sampling protocol (Figures S1 and S8 in ref [1], Table S1 (attached excel file)). The free energy converged after 5–8 ns and no further significant improvement after durations of up to 50 ns was observed (data not shown). We also investigated the output trajectories. After execution of two independent 10 ns simulations the PFuDA ligand adopted a conformation which was closer to that of DA, and the ligand-protein contacts more resembled those in the DA X-ray structure compared to the starting structure Figure S2. As expected, the PFuDA ligand adopted a planar conformation. For the remaining sampling schemes, there were either no reasonable predictions of less than 2.0 $kcal/mol$ or more than one of the simulations was poorly converged. It should be noted that in order to save computational time we did not complete all of these simulations, and for some of them we monitored only the perturbation of DA to PFuDA, Perfluorooctanoic acid (PFOA) and Perfluorohexanoic acid (PFHxA) because the standard FEP+ protocol provided the most significant errors mainly for these transformations. Thus, if we observed data similar to the default FEP+ sampling protocol error, we ended the calculations (data not shown). However, completion of only these perturbations provided us significant information for our protocol development because the main source of the error ($RMSE$ and MUE) was generated by these transformations.

Another critical decision in FEP+ calculations based on the MD-derived structures is whether an averaged structure or those from clustering to be used as a starting system. Structures obtained both via averaged MD and clustering are common in the literature. In our case the preliminary conventional (cMD) and accelerated (aMD) [2] results clearly

demonstrated the presence of multiple binding modes [1].Therefore, the most probable (most frequently present) binding mode can be more accurately obtained by cluster analysis. Moreover, the averaged and minimized structure may eventually "trap" the system into a deeper local energy minimum which presumably explains the requirement for longer pre-REST simulations. We did not perform separate FEP+ analyses for each binding mode as researchers previously suggested [3] because this is beyond the scope of the current study and consider increased pre-REST sampling as an alternative approach for analyzing considerable ligand–protein interactions. Thus, we repeated the aforementioned procedure with the same combination of simulation times using the PFuDA-PPARγ complex in a low-energy minimum (where the PFuDA conformation more closely resembles those of DA) obtained by clustering. A protocol using 5 ns pre-REST sampling and 8 ns REST simulation lead to greater improvement ($RMSE$ = 1.23, R^2 = 0.9; (Figure 7A and 7B in ref [1]).

Based on these results we suspect that the long (2×10 ns) and short (5 ns) pre-REST samplings perform different functions and have different applications. The longer simulation protocol is more suitable to systems when major conformational changes are expected (as in PFuDA average structure), whereas the shorter simulation protocol is more suitable for either lesser conformational changes or regular systems (ligands that more closely resemble the X-ray structure). In addition to the exception of the aforementioned complexity (large and flexible LBD), PPARγ is also a difficult target for some ligand sets for several reasons. For instance, dimerization in the nuclear receptors is an important factor for LBD conformation, in particular helix 12 [1][4][5][6]. In the native state PPAR dimerizes with the retinoid X receptor (R×R) but forms a homodimer in crystallographic experiments. The dimerization interface is situated at helices 6, 7, and 11, which surrounds and modulates the PFuDA binding site, rendering MD simulations in the monomer state less realistic for long-chain ligands that bind to the same cavity. In addition, during the our preliminary MD simulations the transformation of PFuDA from an initial orientation, as obtained via a docking procedure in the Rosi-PPARγ X-ray structure, to those similar to DA introduced considerable changes in the conformation of residues in the LBD [1]. This also affected the mobility of small-chain ligands, their realistic ligand-receptor interactions, and their predicted ΔG values as per FEP+.

Nonetheless, even in this case we clearly showed that more-intensive equilibration per lambda during the pre-REST step considerably improved the $\Delta\Delta G$ values for all of the perturbations. The correct ranking of all ligands (6/6), the good correlation coefficient (R^2 = 0.9), and an $RMSE$ error of 1.2 $kcal/mol$ are reasonable results and may be helpful during the lead optimization process. Thus we decided to test our sampling protocol on several other systems and compare results to the default FEP+ protocol.

2 Tables

Table 1: The total number of atoms (N of atoms), number of waters (N of water), number of Ions (N of Ions), Box dimensions (x, y, z) and the charge of the ligands for each system in this study.

System	N of atoms	N of water	N of Ions	Box dimensions	Charge
T4 lysozyme	24276	21591	50	58.9; 62.2 71.5	0
AKT1	38412	32925	70	69.4; 70.2; 85.3	+1
THR	35000	30279	56	69.3; 69.3; 77.6	+1
TYK2	37004	32271	63	68.8; 68.9; 83.6	0
PPARgamma	35174	30675	63	74.2; 77.4; 65.8	-1

3 Employed scripts

For the transformations in complex with protein the following script modifications were used in the FEP+ *.msj file (Schrodinger software version 2017-3):

```
simulate {
  effect_if = [
   ["==" -gpu "@*.*.jlaunch_opt[-1]" ]
   "ensemble.method = Langevin"
  ]
  eneseq = {
    interval = 0.3
  }
  ensemble = {
    barostat = {
      tau = 2.0
    }
    class = NPT
    method = Berendsen
    thermostat = {
      tau = 0.1
    }
  }
  randomize_velocity = {
    first = 0.0
  }
  time = 10000
  title = "NPT and no restraints, second 10ns"
  trajectory = {
    center = solute
    interval = 240.0
  }
}
```

```
lambda_hopping {
  backend = {
    migration.interval = 0.024
  }
  checkpt = {
    write_last_step = true
  }
  energy_group = {
    first = 0.0
    interval = 1.2
    name = "$JOBNAME$[_replica$REPLIstrCA$]_enegrp.dat"
  }
  ensemble = NPT
  fep_convergence = 0.0
  randomize_velocity = {
    first = 0.0
    interval = inf
    seed = 2007
    temperature = "@*.temperature"
  }
  solute_tempering = {
    atom = "asl: res.ptype SMH "
    temperature = {
      exchange_probability = 0.3
      generator = PvdS
    }
  }
  time = 8000.0
  timestep = [0.004 0.004 0.008 ]
  title = "NPT, 8000ps"
  trajectory = {
    center = solute
    interval = 240.0
  }
}
```

4 Figures

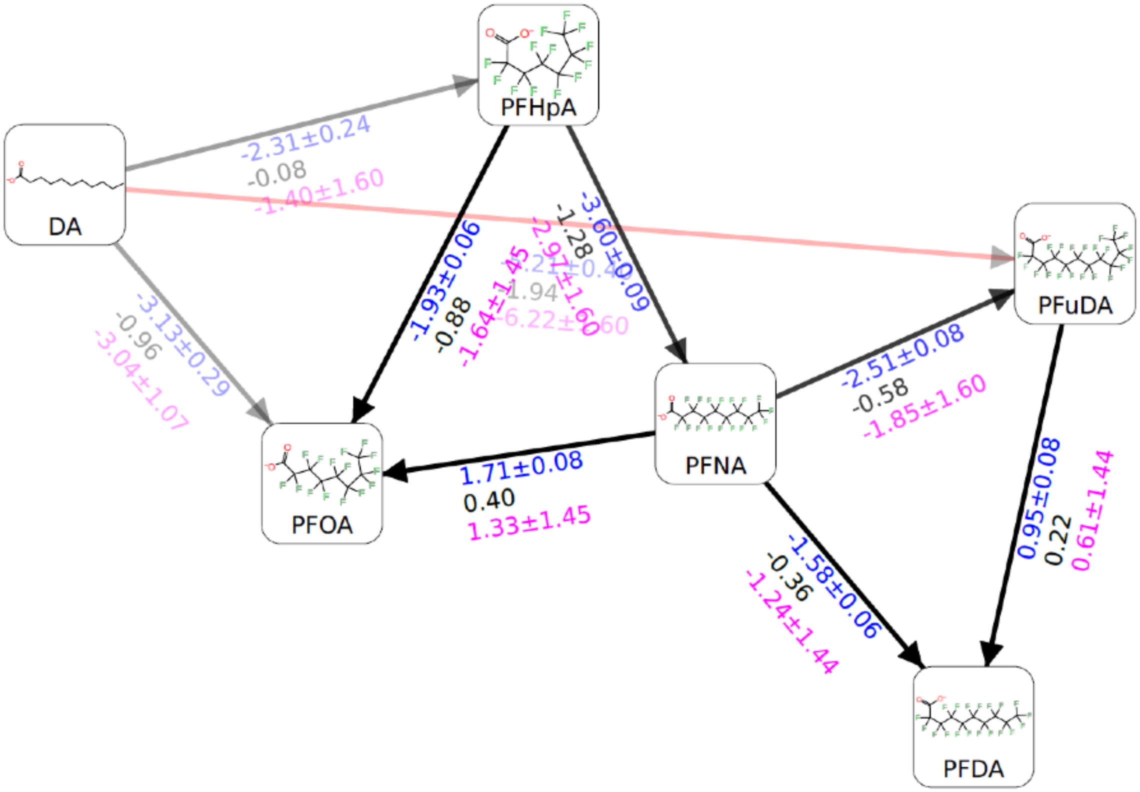

Figure 1: Free energy output map obtained via the 2×10 *ns* pre-REST FEP+ sampling protocol for the PPARγ set of ligands. Black, blue, and red indicate the experimental ($\Delta\Delta G_{exp}$), calculated Bennett ($\Delta\Delta G_{pred}$), and cycle closure ($\Delta\Delta G_{pred_c}$) free energies of binding, respectively

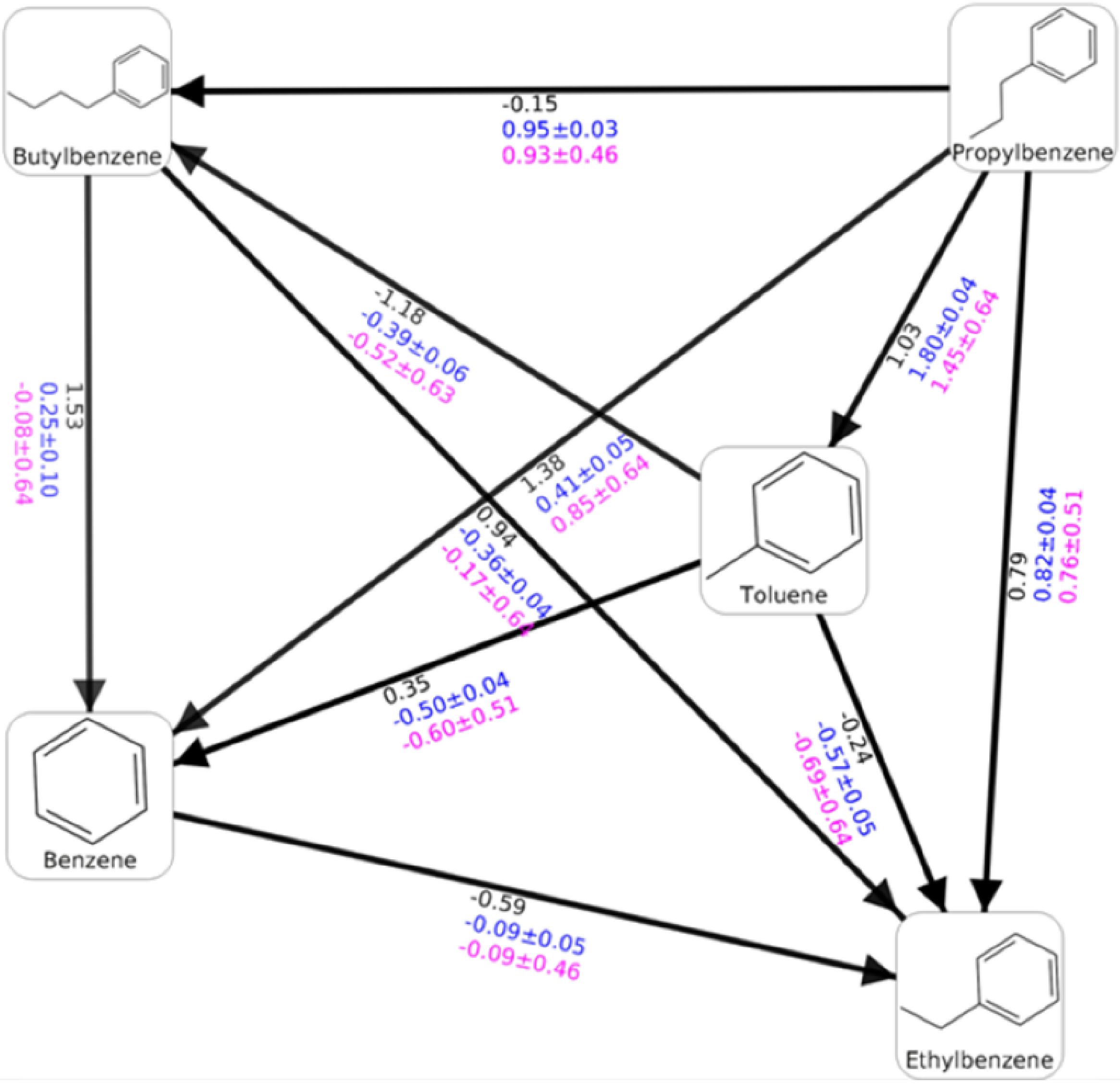

Figure 2: Free energy output map obtained via the 2×10 ns pre-REST FEP+ sampling protocol for the T4 lysozyme L99A set of ligands for which experimental $\Delta\Delta G$ values are available. Black, blue, and red indicate the experimental ($\Delta\Delta G_{exp}$), calculated Bennett ($\Delta\Delta G_{pred}$), and cycle closure ($\Delta\Delta G_{pred_c}$) free energies of binding, respectively

Solvent Leg

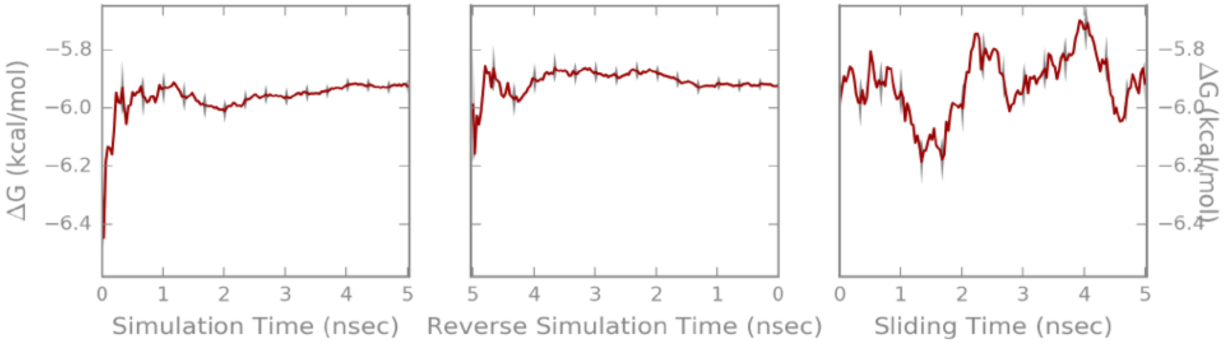

λ pair:	0,1	1,2	2,3	3,4	4,5	5,6	6,7	7,8	8,9	9,10	10,11	Total
Forward dF:	-1.15	-0.91	-0.76	-0.66	-0.42	-0.76	-0.46	-0.23	-0.16	-0.25	-0.15	-5.93
Bootstrap STD:	0.006	0.006	0.006	0.005	0.007	0.010	0.010	0.007	0.008	0.006	0.006	0.024
Analytical STD:	0.006	0.006	0.005	0.005	0.009	0.010	0.009	0.007	0.006	0.006	0.007	0.024

Complex Leg

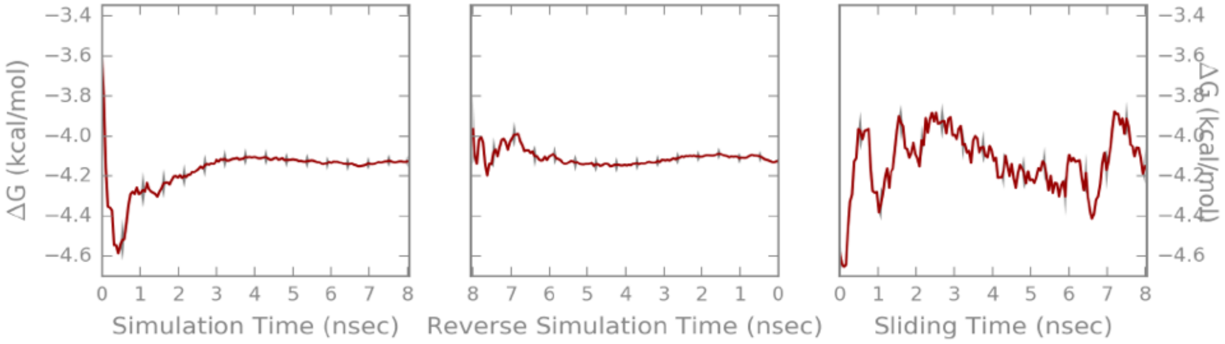

Figure 3: Observed convergence during simulation of the propylbenzene→toluene ligand transformation in T4 lysozyme L99A.

Solvent Leg

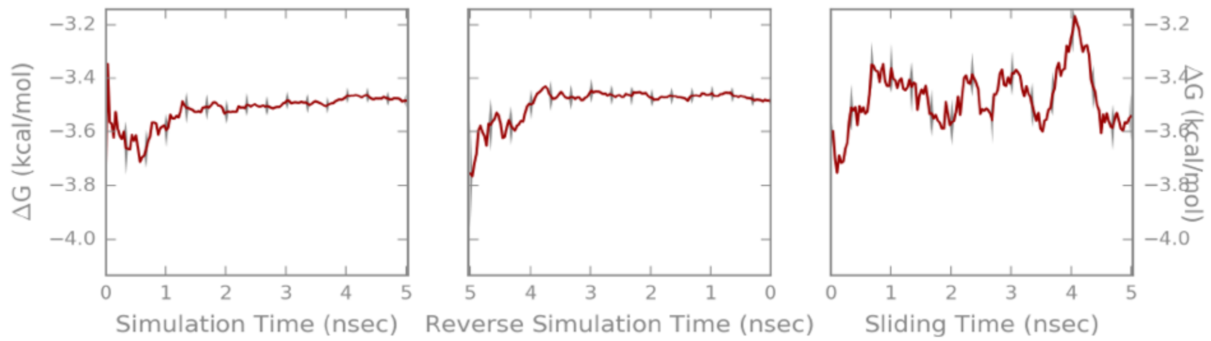

λ pair:	0,1	1,2	2,3	3,4	4,5	5,6	6,7	7,8	8,9	9,10	10,11	Total
Forward dF:	-1.38	-0.88	-0.61	-0.44	-1.73	-1.10	0.02	0.12	0.47	0.66	1.39	-3.48
Bootstrap STD:	0.008	0.006	0.004	0.004	0.013	0.009	0.010	0.006	0.008	0.005	0.007	0.026
Analytical STD:	0.006	0.006	0.005	0.005	0.011	0.012	0.011	0.008	0.007	0.007	0.007	0.026

Complex Leg

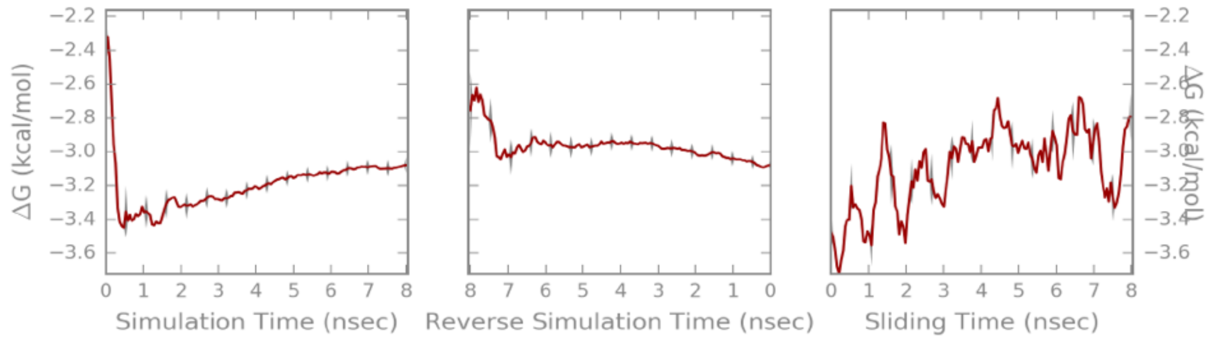

Figure 4: Observed convergence during simulation of the propylbenzene→benzene ligand transformation in T4 lysozyme L99A, demonstrating the need of extension of the REST simulation time in our 2×10 ns sampling protocol to at least 8 ns.

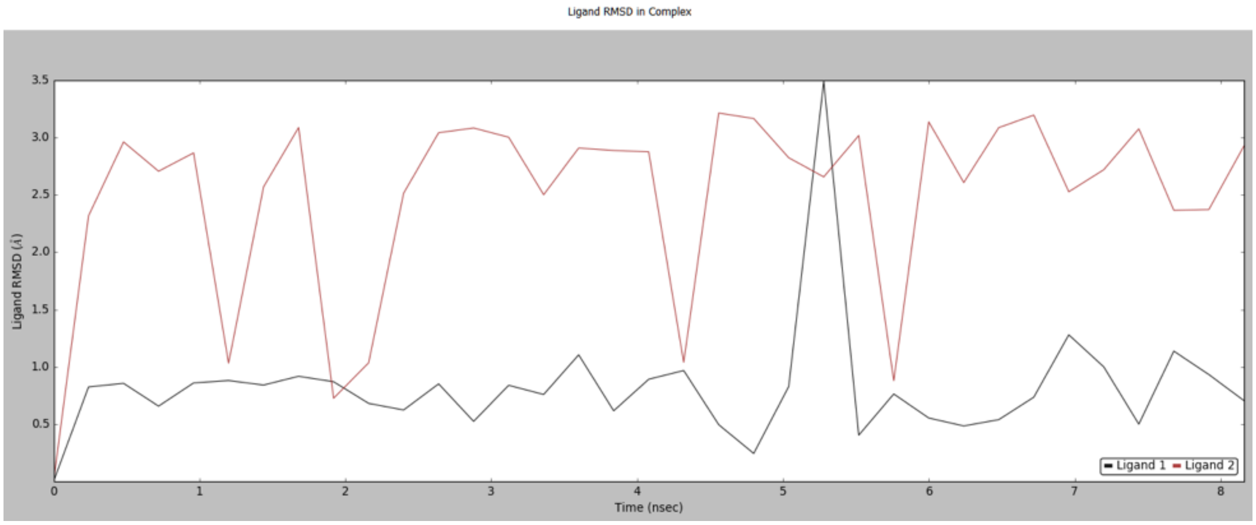

Figure 5: Observed ligand root mean squared deviation ($RMSD$) changes in T4 lysozyme L99A during the course of REST simulations of propylbenzene to butylbenzene transformation. Ligand 1 is propylbenzene whereas Ligand 2 is butylbenzene

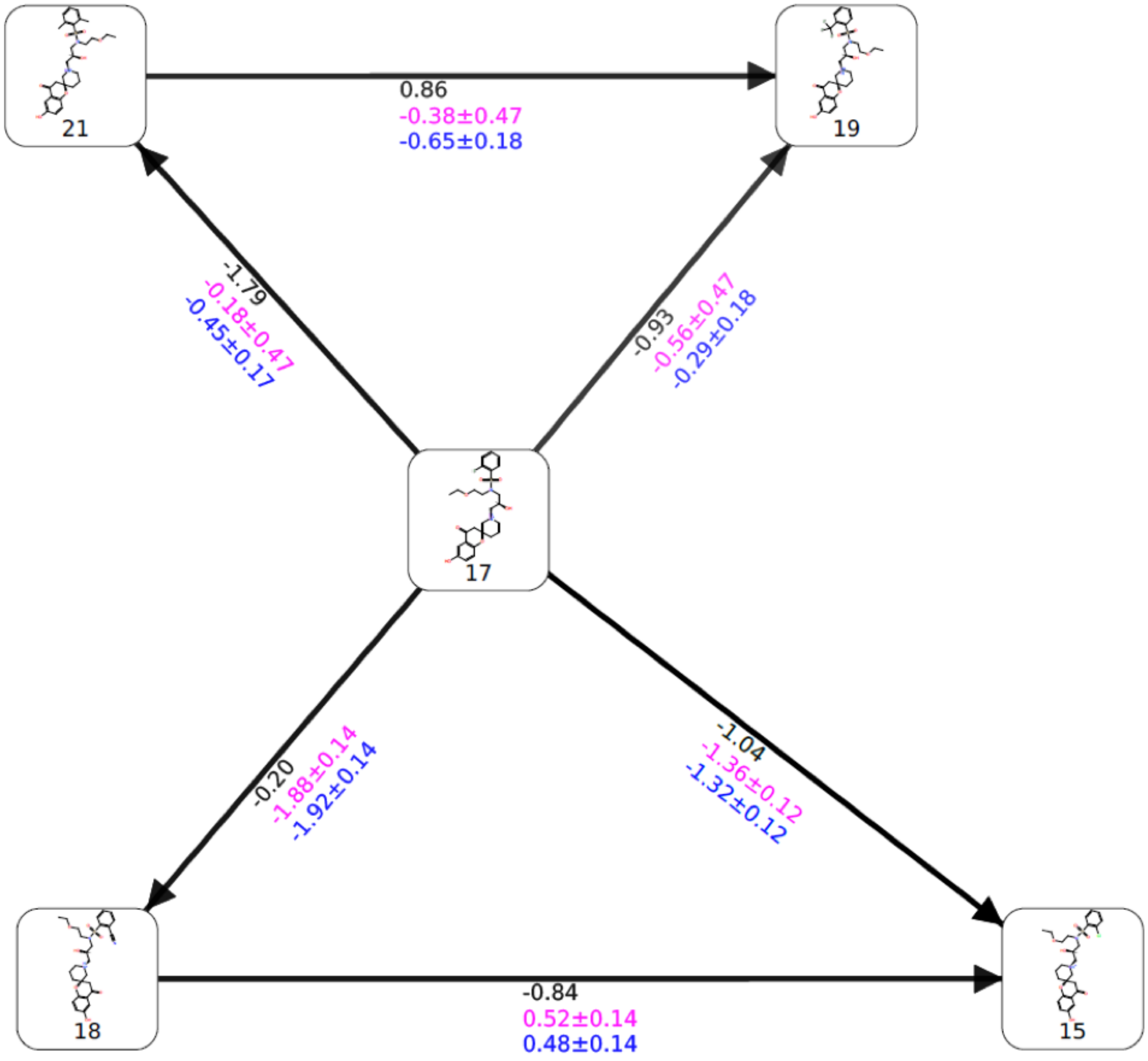

Figure 6: Free energy output map obtained via the default FEP+ sampling protocol for the small AKT1 test set of ligands. Black, blue, and red indicate the experimental ($\Delta\Delta G_{exp}$), calculated Bennett ($\Delta\Delta G_{pred}$), and cycle closure ($\Delta\Delta G_{pred_c}$) free energies of binding, respectively

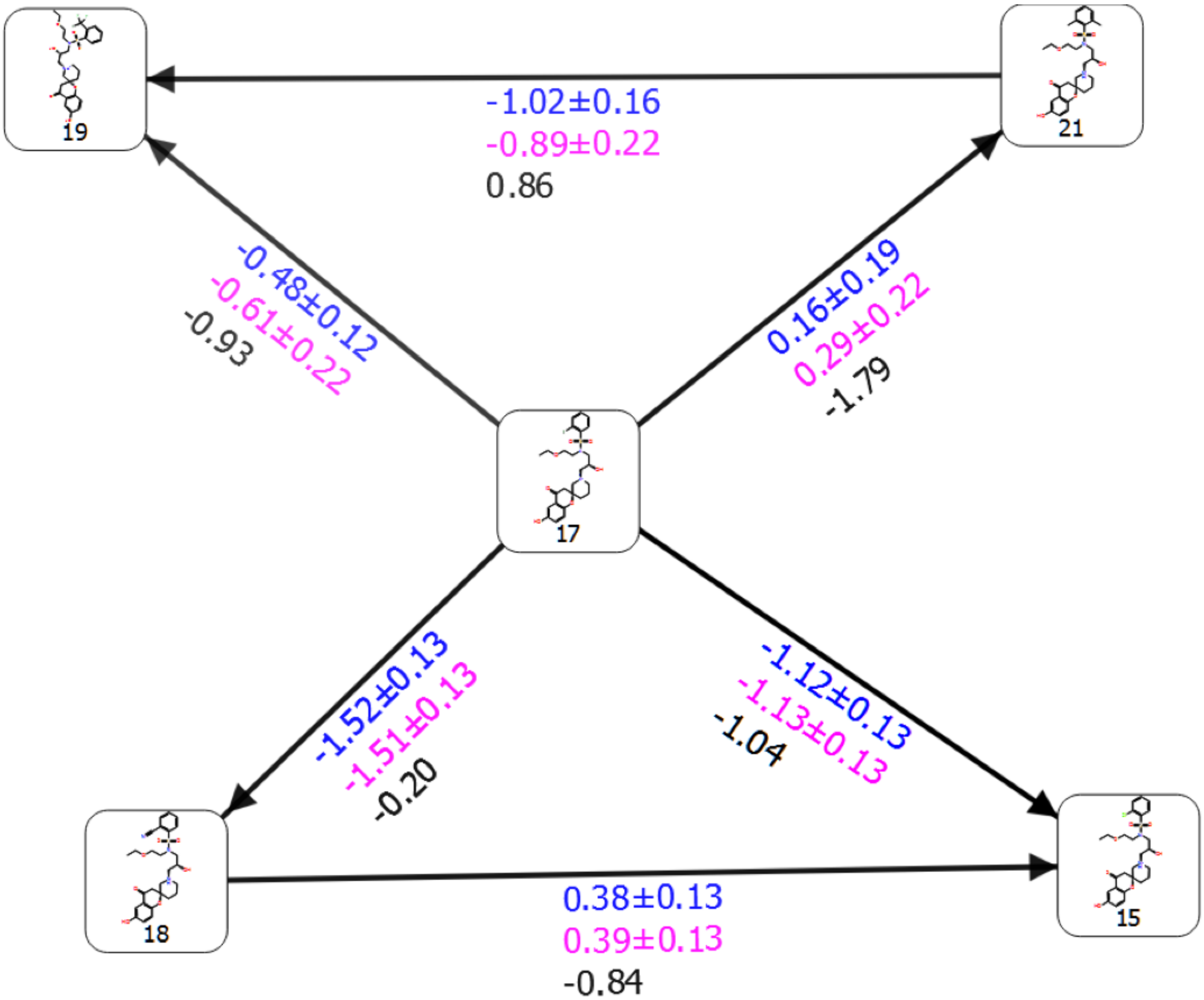

Figure 7: Free energy output map obtained via the pREST FEP+ sampling protocol, in which Gly159, Phe161, and Gly162 were included in the ”hot region” for the small AKT1 test set of ligands. Black, blue, and red indicate the experimental ($\Delta\Delta G_{exp}$), calculated Bennett ($\Delta\Delta G_{pred}$), and cycle closure ($\Delta\Delta G_{pred_c}$) free energies of binding, respectively.

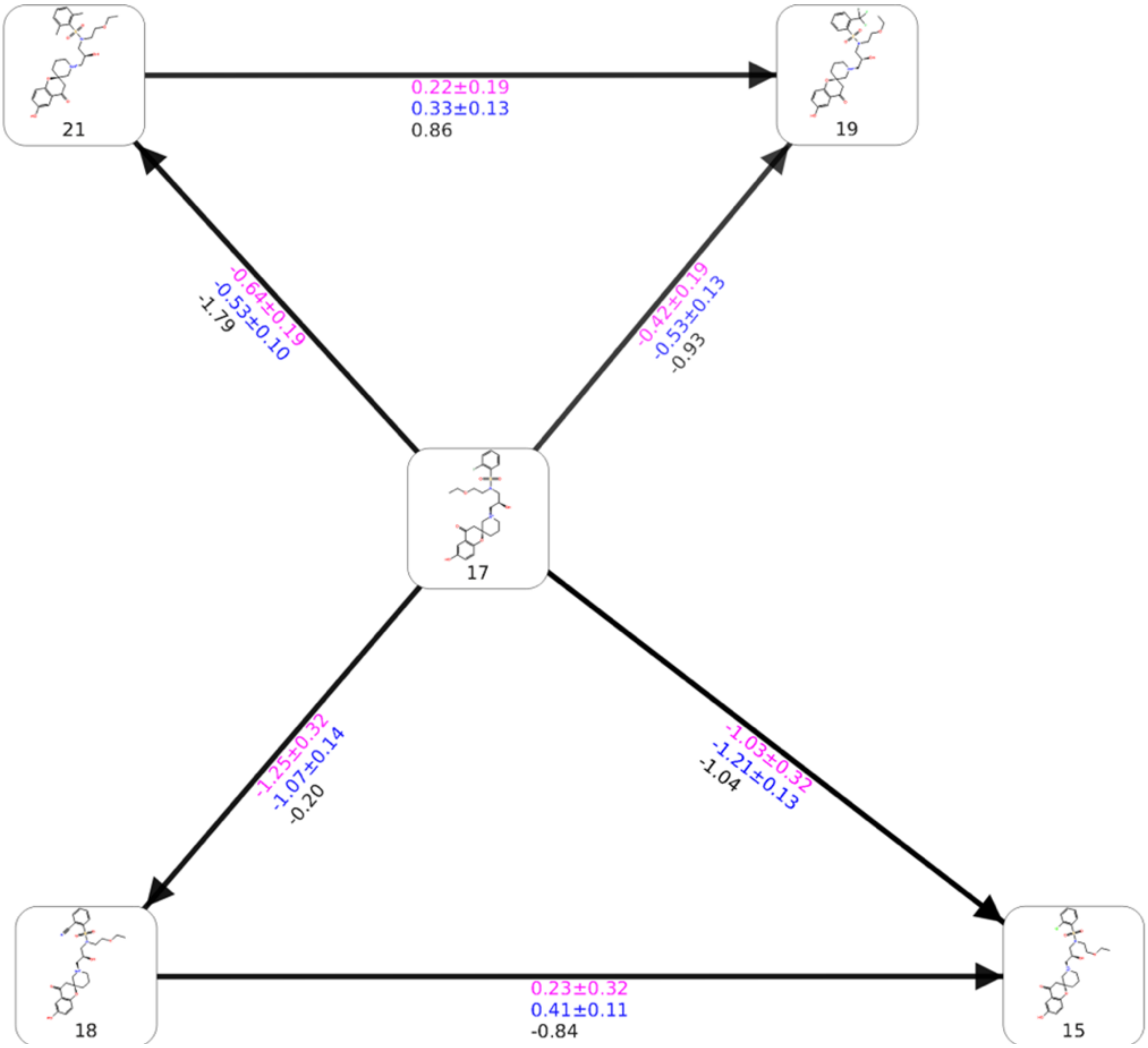

Figure 8: Free energy output map obtained via our 5 ns pre-REST FEP+ sampling protocol for the small AKT1 test set of ligands. Black, blue, and red indicate the experimental ($\Delta\Delta G_{exp}$), calculated Bennett ($\Delta\Delta G_{pred}$), and cycle closure ($\Delta\Delta G_{pred_c}$) free energies of binding, respectively.

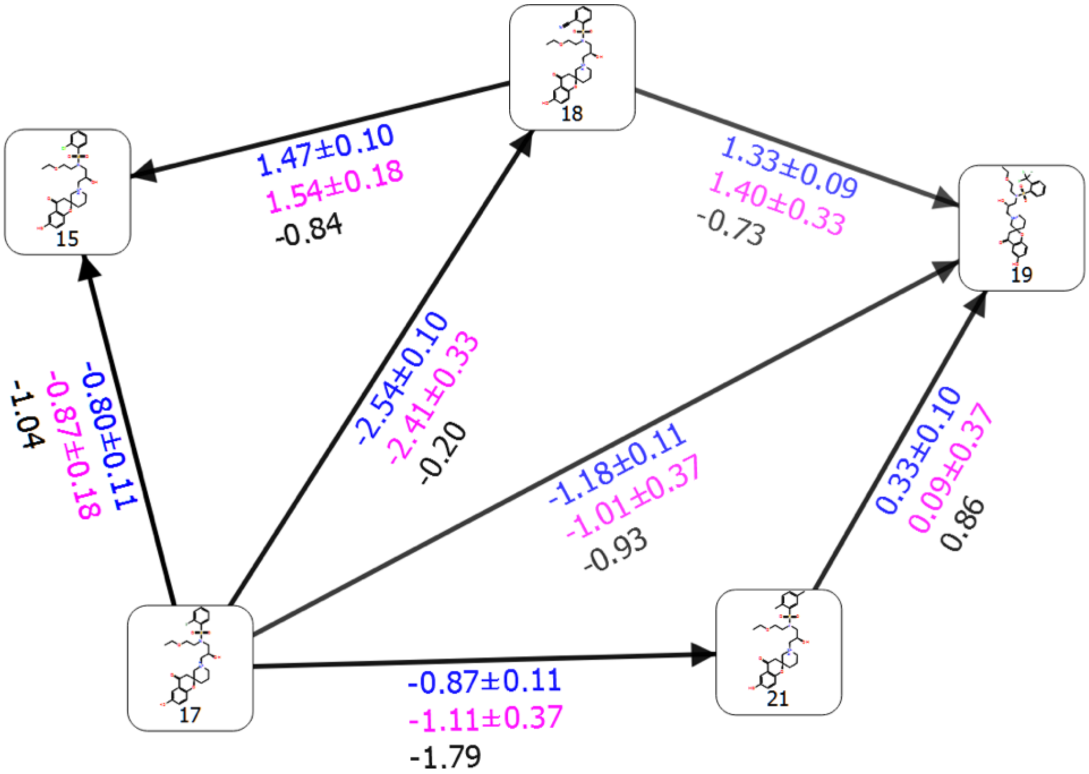

Figure 9: Free energy output map obtained via an averaged AKT1 MD structure in a complex with ligand 19 and our 5 ns pre-REST FEP+ sampling protocol for the small AKT1 test set of ligands. Black, blue, and red indicate the experimental ($\Delta\Delta G_{exp}$), calculated Bennett ($\Delta\Delta G_{pred}$), and cycle closure ($\Delta\Delta G_{pred_c}$) free energies of binding, respectively

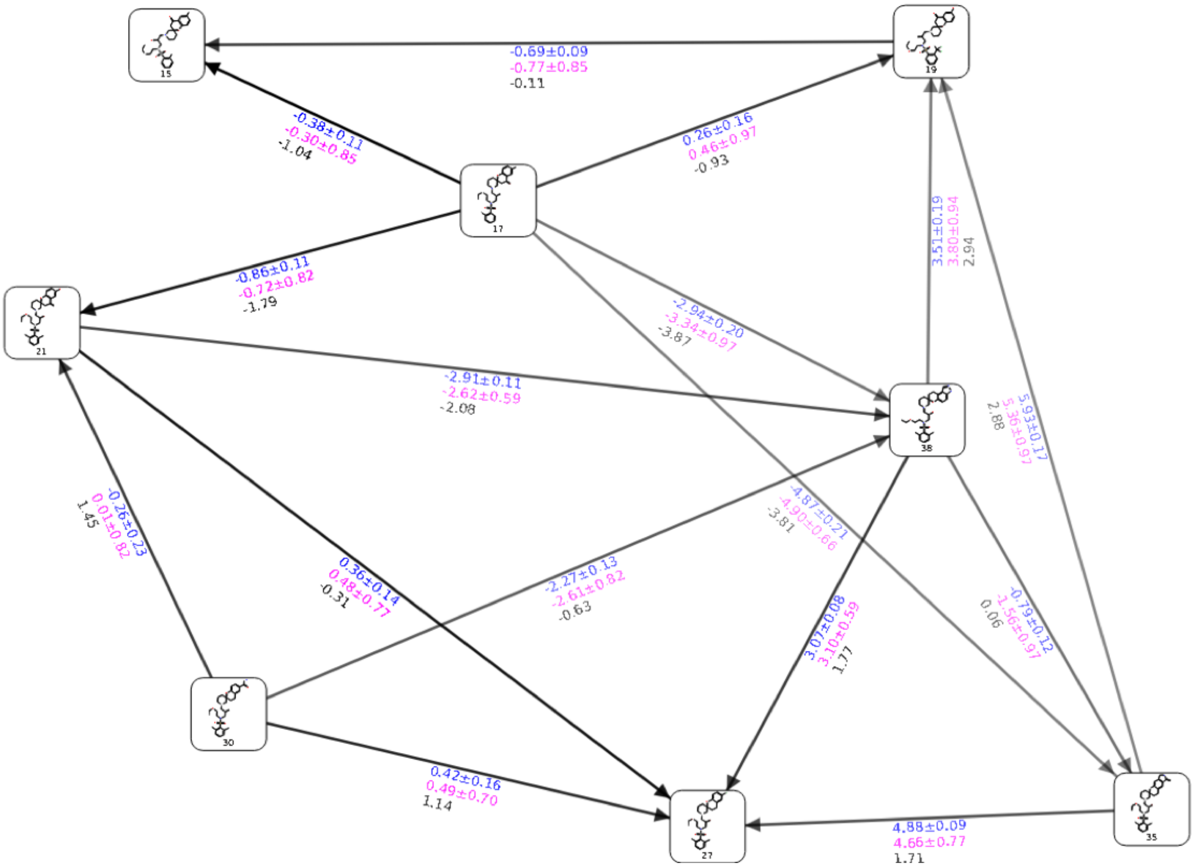

Figure 10: Free energy output map obtained via the default FEP+ sampling protocol for all of the included AKT1 test set of ligands and perturbations. Black, blue, and red indicate the experimental ($\Delta\Delta G_{exp}$), calculated Bennett ($\Delta\Delta G_{pred}$), and cycle closure ($\Delta\Delta G_{pred_c}$) free energies of binding, respectively.

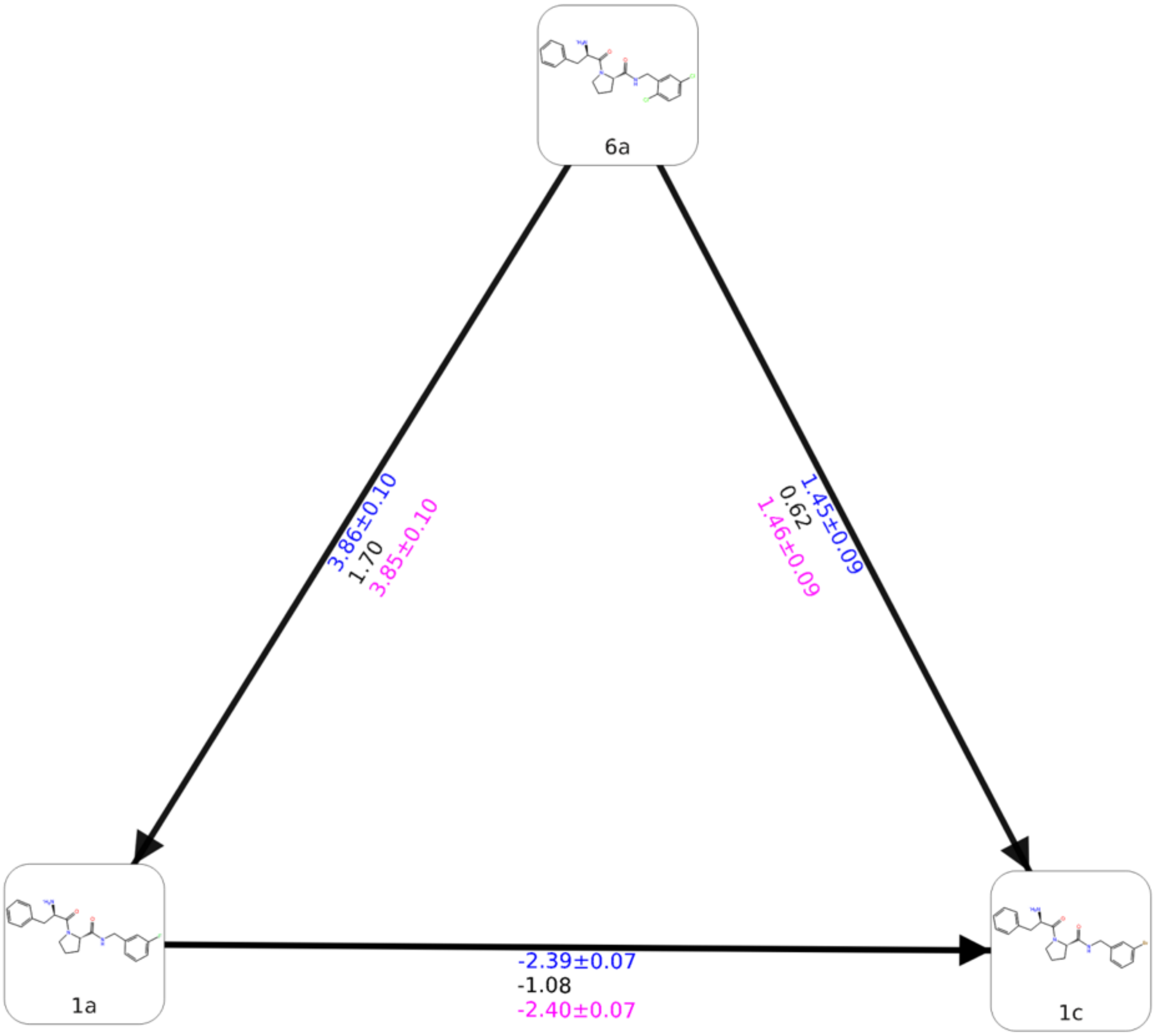

Figure 11: Free energy output map obtained via the default FEP+ sampling protocol for the selected small THR test set of ligands and perturbations. Black, blue, and red indicate the experimental ($\Delta\Delta G_{exp}$), calculated Bennett ($\Delta\Delta G_{pred}$), and cycle closure ($\Delta\Delta G_{pred_c}$) free energies of binding, respectively.

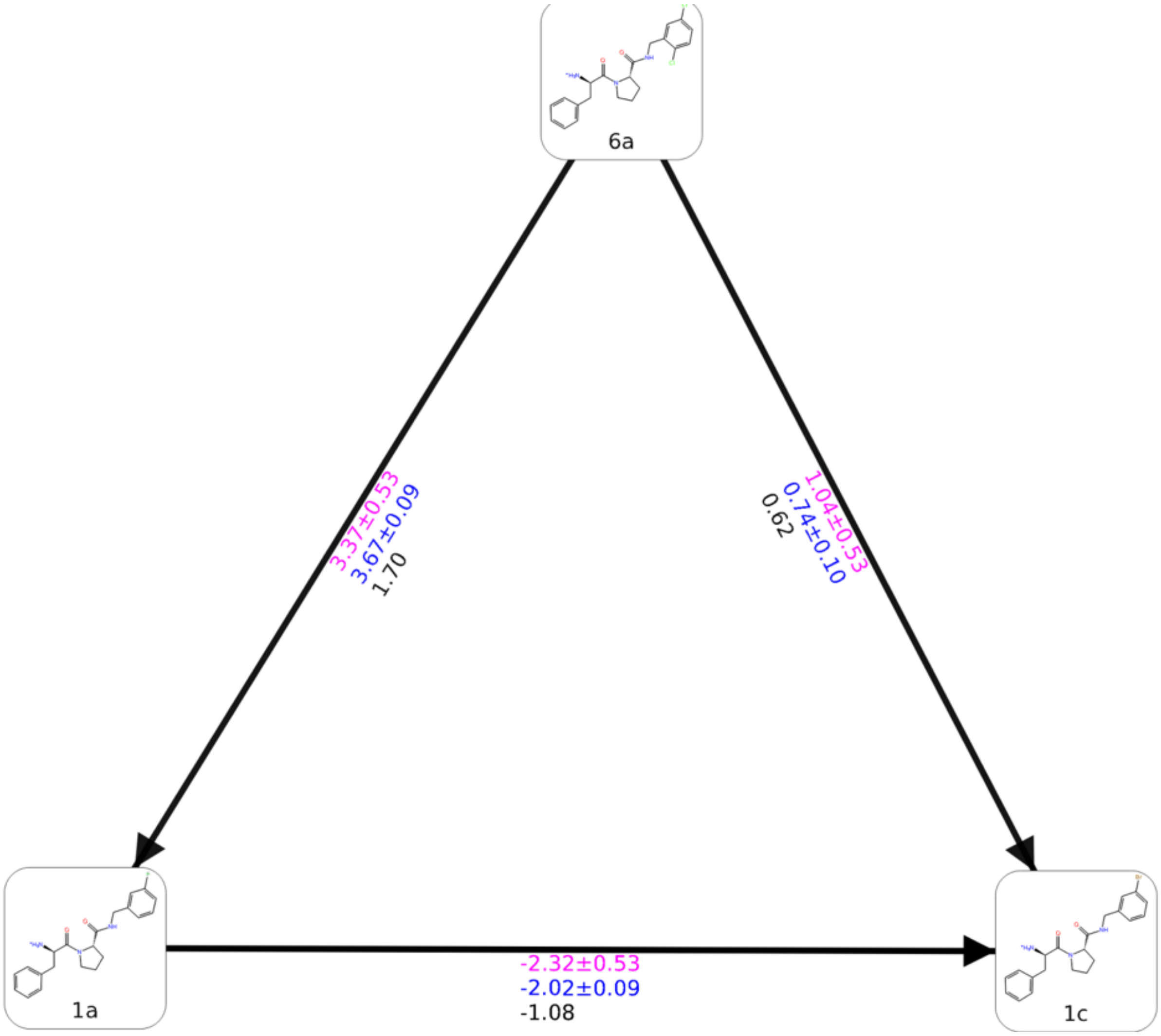

Figure 12: Free energy output map obtained via our 5 ns pre-REST FEP+ sampling protocol for the selected small THR test set of ligands and perturbations. Black, blue, and red indicate the experimental ($\Delta\Delta G_{exp}$), calculated Bennett ($\Delta\Delta G_{pred}$), and cycle closure ($\Delta\Delta G_{pred_c}$) free energies of binding, respectively.

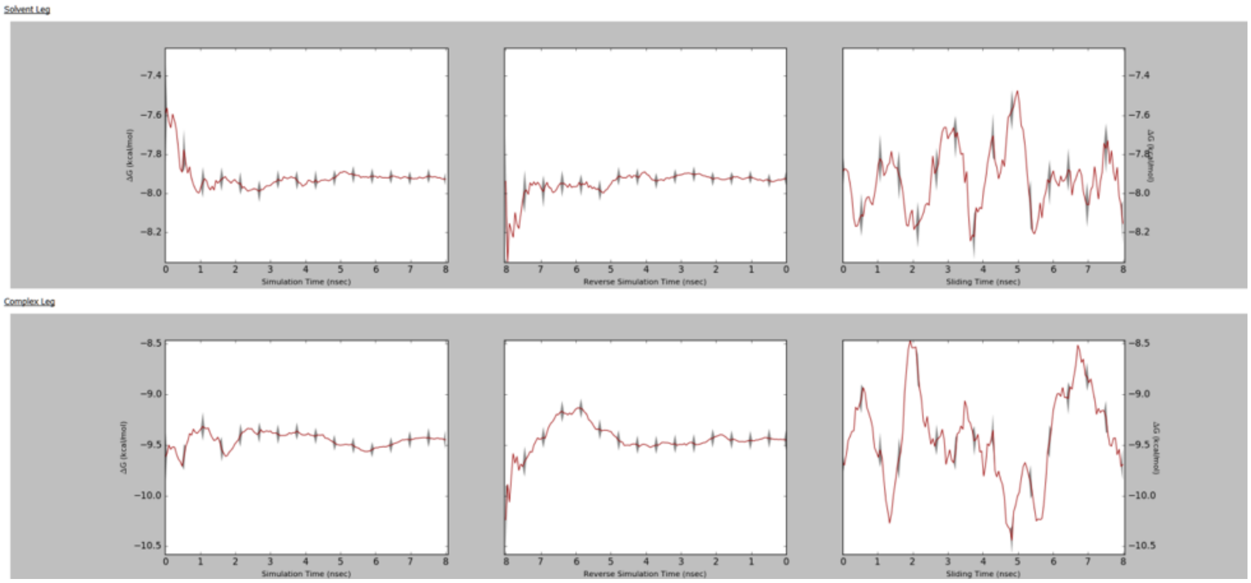

Figure 13: Examples of the observed changes in the free energies during the simulation, reversion, and slide times, showing the convergence of the 1d→6e ligand transformation from the THR ligand test set.

Solvent Leg

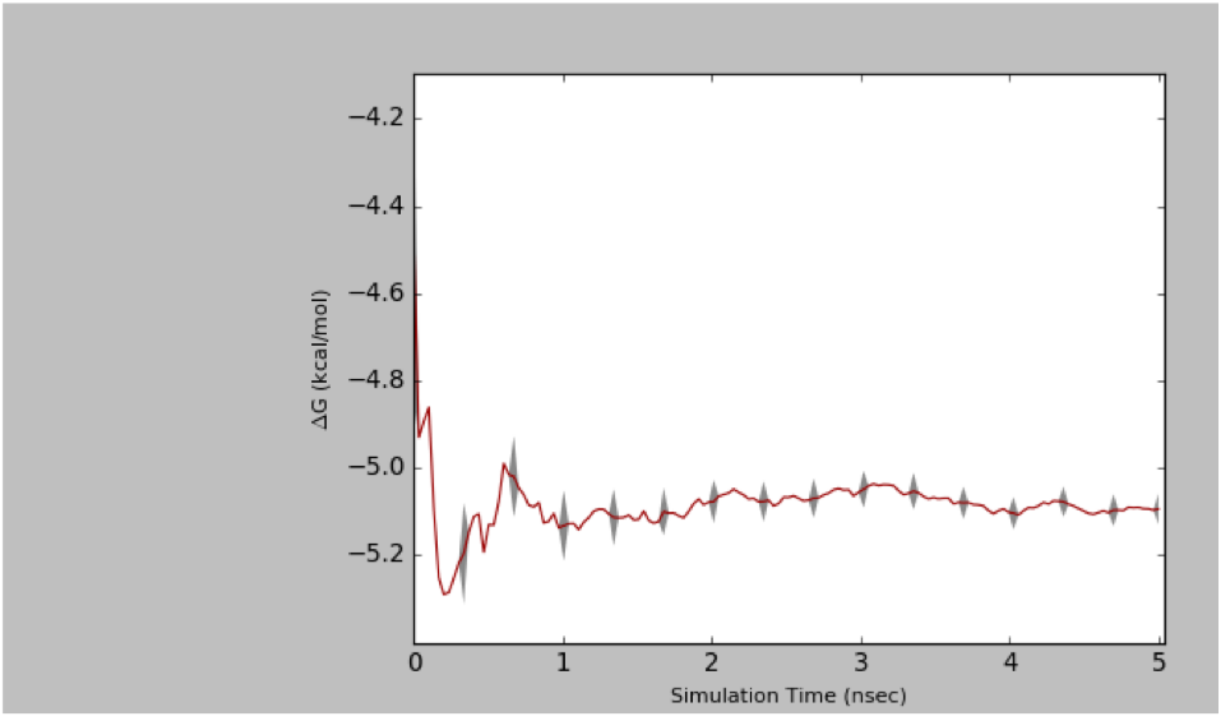

Complex Leg

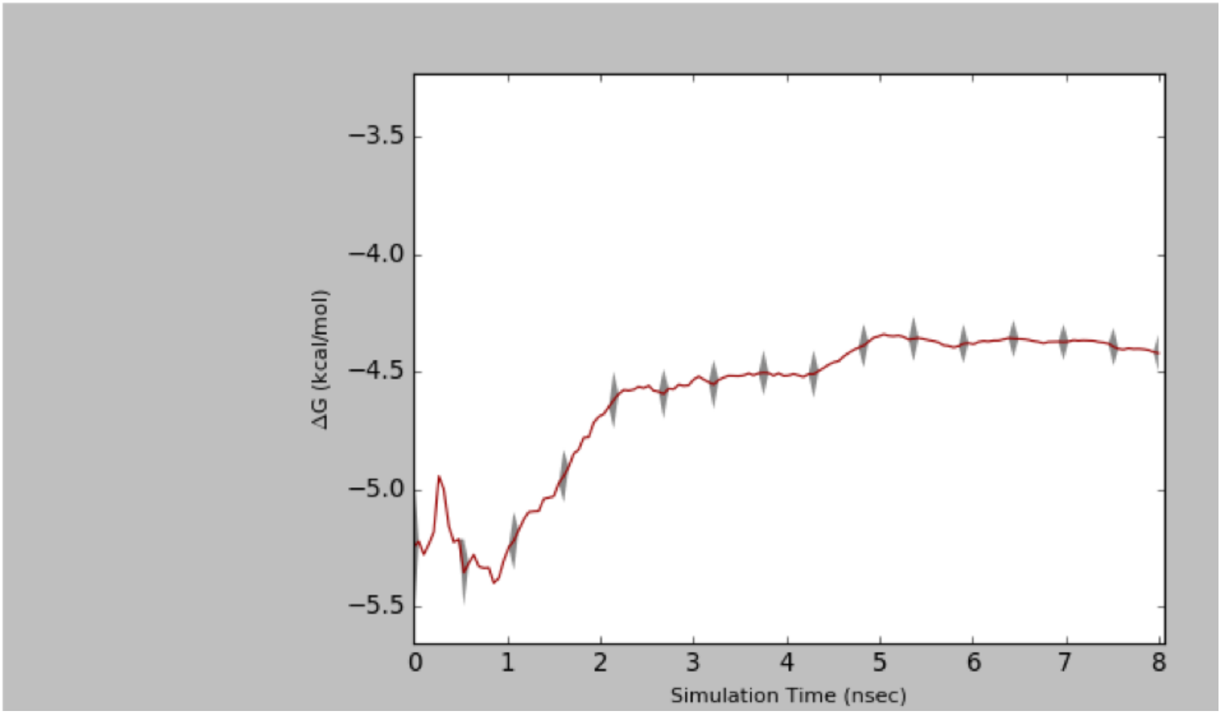

Figure 14: Free energies during simulation of the 1b→7a ligand transformation (convergence), demonstrating that improved results were not obtained due to extension of the REST simulation.

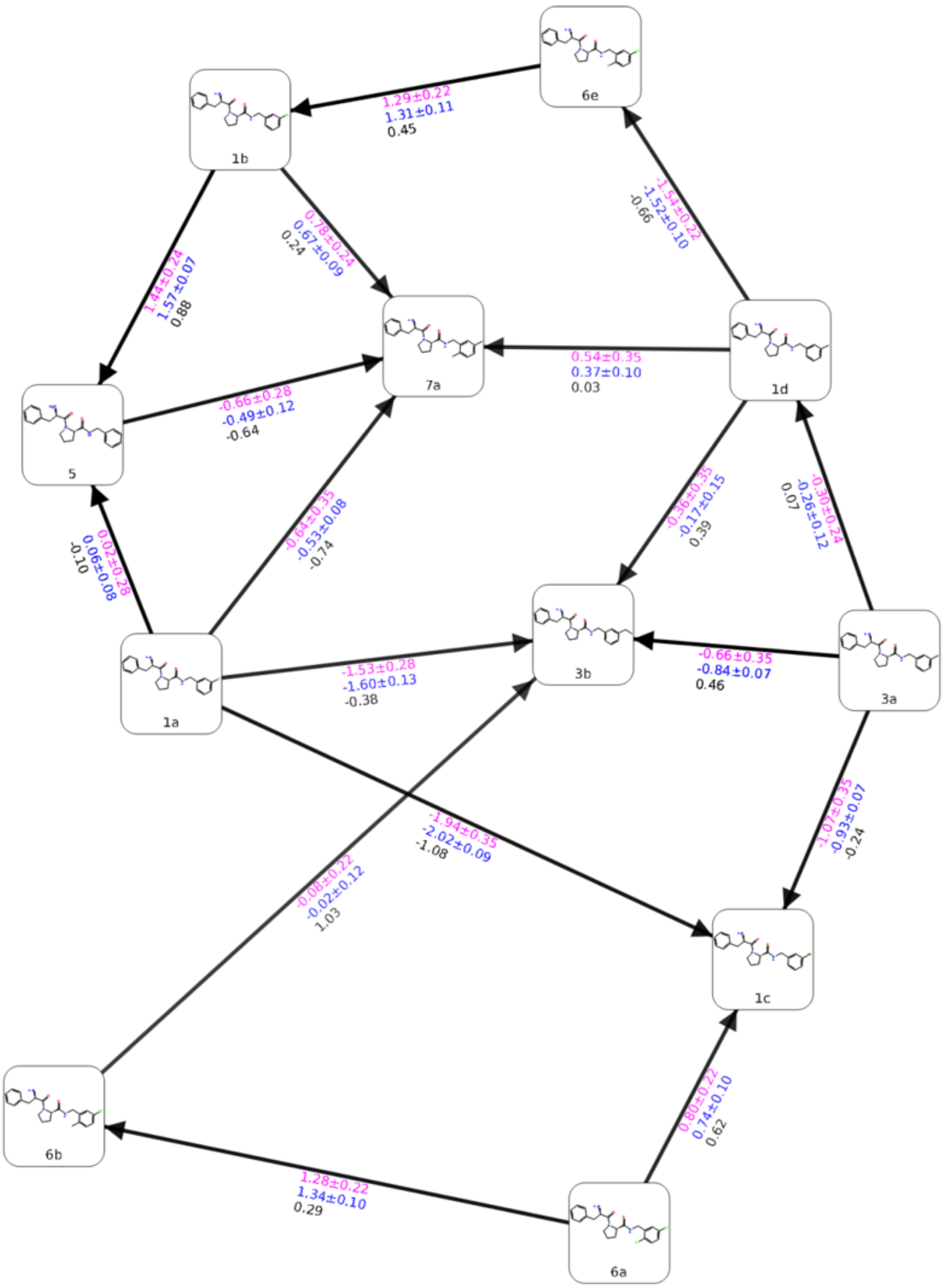

Figure 15: Free energy output map obtained via our 5 ns pre-REST FEP+ sampling protocol and transformations created via default LOMAP for the selected THR test set of ligands and perturbations. Black, blue, and red indicate the experimental ($\Delta\Delta G_{exp}$), calculated Bennett ($\Delta\Delta G_{pred}$), and cycle closure ($\Delta\Delta G_{pred_c}$) free energies of binding, respectively.

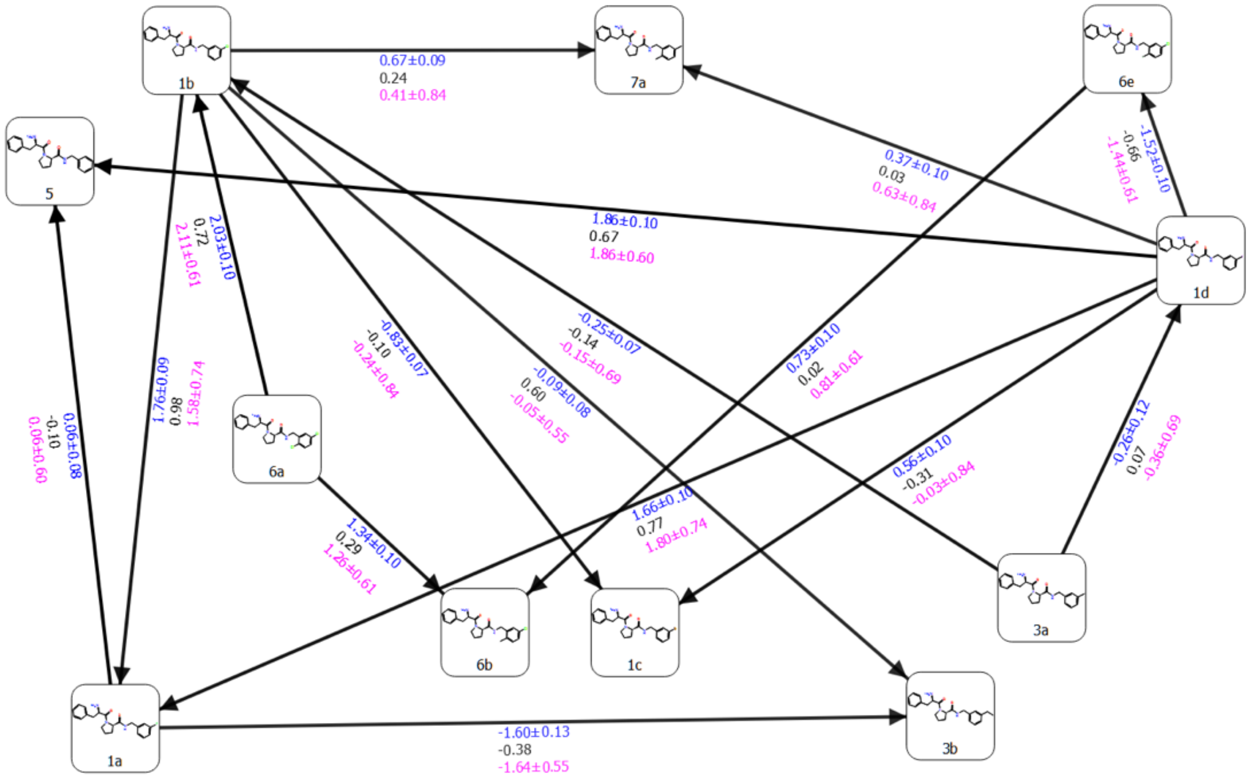

Figure 16: Free energy output map obtained via our 5 ns pre-REST FEP+ sampling protocol for the selected THR test set of ligands and all executed transformations. Black, blue, and red indicate the experimental ($\Delta\Delta G_{exp}$), calculated Bennett ($\Delta\Delta G_{pred}$), and cycle closure ($\Delta\Delta G_{pred_c}$) free energies of binding, respectively.

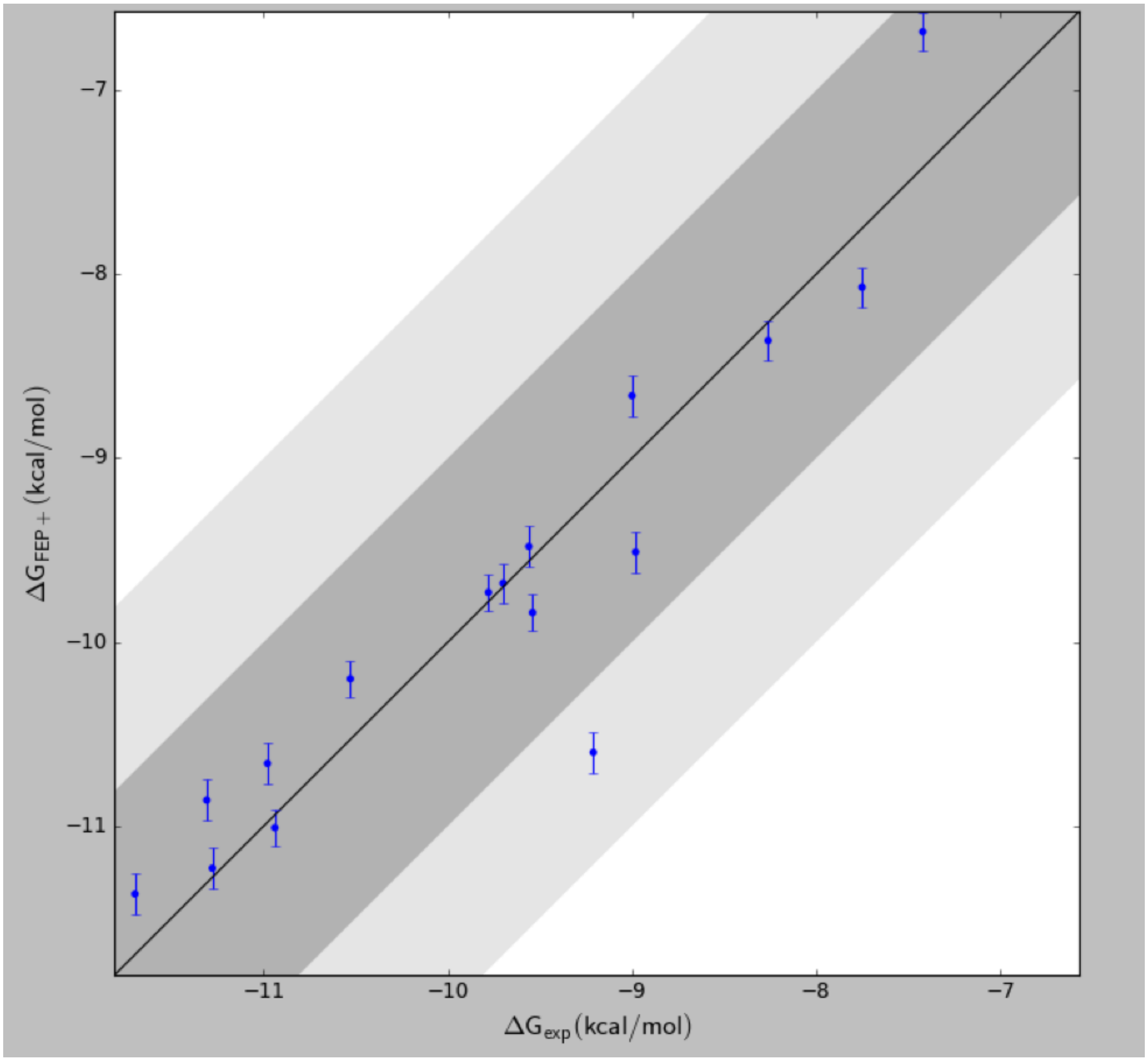

Figure 17: Regression obtained between the experimental and predicted free energies for selected TYK2 test set of ligands.

References

[1] Filip Fratev, Steinbrecher Thomas, and Jónsdóttir Svava Ósk. Prediction of accurate binding modes using combination of classical and accelerated molecular dynamics and free-energy perturbation calculations: An application to toxicity studies. *ACS Omega*, 3(4):4357–4371, 2018.

[2] D Hamelberg, J Mongan, and J. A. McCammon. Accelerated molecular dynamics: A promising and efficient simulationmethod for biomolecules. *J .Chem. Phys.*, 120(24):11919–11929, 2004.

[3] Joseph W. Kaus, Edward Harder, Teng Lin, Robert Abel, J. Andrew McCammon, and Lingle Wang. How to deal with multiple binding poses in alchemical relative protein–ligand binding free energy calculations. *Journal of Chemical Theory and Computation*, 11(6):2670–2679, 2015.

[4] Filip Fratev, Ivanka Tsakovska, Merilin Al Sharif, Elina Mihaylova, and Ilza Pajeva. Structural and dynamical insight into ppar antagonism: In silico study of the ligand-receptor interactions of non-covalent antagonists. *International Journal of Molecular Sciences*, 16(12):15405–15424, Jul 2015.

[5] Filip Fratev. Activation helix orientation of the estrogen receptor is mediated by receptor dimerization: evidence from molecular dynamics simulations. *Phys. Chem. Chem. Phys.*, 17:13403–13420, 2015.

[6] Filip Fratev. Pparγ helix 12 exhibits an antagonist conformation. *Phys. Chem. Chem. Phys.*, 18:9272–9280, 2016.
